# Manualised Attachment-Based Interventions for Improving Caregiver-Infant Relationships: A Two-Stage Systematic Review

**DOI:** 10.1007/s10567-024-00497-0

**Published:** 2024-11-18

**Authors:** A. Wittkowski, C. Crompton, M. W. Wan

**Affiliations:** 1 School of Health Sciences, The University of Manchester, Manchester, UK; 2 Greater Manchester Mental Health NHS Foundation Trust, Manchester, UK; 3 Manchester Academic Health Science Centre, Manchester, UK; 4 Division of Psychology and Mental Health, School of Health Sciences, Faculty of Biology, Medicine and Health, Manchester Academic Health Science Centre, The University of Manchester, Oxford Road, 2nd Floor Zochonis Building, Room 2.41, Manchester, M13 9PL UK

**Keywords:** Attachment, Parent, Child, Infant, Perinatal, Intervention effectiveness

## Abstract

As attachment-based interventions can improve caregiver-infant relationships and their subsequent psychological outcomes, the identification of relevant and effective interventions can facilitate their implementation into clinical practice. This systematic review aimed to a) provide an overview of manualised attachment-based interventions, without video-feedback as the main component, for caregivers and infants from conception to two years, and b) determine which of these interventions were effective in demonstrating improvements in caregiver-infant relational outcomes. To identify eligible interventions and their empirical evidence base, two search stages were conducted for 1) relevant interventions and 2) studies of interventions identified in the first stage that focussed on caregiver-infant relational outcomes. All studies included in Stage 2 were quality assessed and findings analysed. Twenty-six interventions were eligible for inclusion at Stage 1 but studies reporting on relational outcomes were identified for 16 interventions only. Forty studies reporting on those 16 interventions met inclusion criteria and were synthesised at Stage 2. Most studies were of good quality. Observer-rated measures were used in 90% of studies. There was evidence for these interventions in relation to improving caregiver-infant relational outcomes: 80% of studies reported a statistically significant positive change in a relational outcome for the intervention compared to pre-intervention or control group. The most promising evidence was identified for *Attachment and Biobehavioral Catch-Up (ABC), Minding the Baby (MTB)* and *Circle of Security (COS)*. This systematic review offers guidance to healthcare professionals, commissioners and policymakers within perinatal sectors in relation to the training, delivery and implementation of evidenced manualised attachment-based interventions.

**Supplementary Information:**

The online version contains supplementary material available at 10.1007/s10567-024-00497-0.

## Introduction

It is widely recognised that caregiver-infant relationships from conception to age two are vitally important for child development. During these early years, infant interactions with emotionally available and sensitive caregivers can have a significant effect on infant outcomes, including attachment security, emotion regulation, confidence to explore the world, learning how to connect and interact with others and brain development (Ainsworth & Bell, 1970; Bowlby, 2005; Cabrera et al., 2017; Cornell & Hamrin, 2008).

Attachment theory provides a framework for understanding that early relational experiences provide an infant with a blueprint for future relationships, including how they relate to themselves and others, as well as providing them with the psychological skills for dealing with adversity (Ainsworth & Bell, 1970; Bowlby, 1969, 1988). Infants who received inconsistent, unpredictable, and emotionally insensitive caregiving throughout their early years have been found to be more at risk of developing negative long-term outcomes (Barlow et al., 2016; Hobson, 2004). The impact of difficulties in early caregiver-infant relationships has also been associated with later mental health difficulties (Catanzaro & Wei, 2010; Doron et al., 2009; Illing et al., 2010; Lyons-Ruth et al., 2013) and physical health difficulties (Anno et al., 2015; McWilliams & Bailey, 2010) in the child.

The significance of early caregiving relationships led to the development of various specialised attachment-based interventions (Kerr & Cossar, 2014). These interventions target the relational processes between caregivers and their infants, including their level of sensitivity, attunement or responsiveness or the dyad’s quality of interactive behaviour, all of which can bring lasting improvements to the caregiver-infant relationship (National Institute of Care Excellence [NICE], 2015; Parent-Infant Foundation, 2023). Improvements may be achieved through direct work with the dyad or with small groups of caregivers, using approaches such as behavioural strategies, mentalisation, video-feedback, psychoeducation, or a mixture of intervention approaches.

The evidence for attachment-based interventions targeting the caregiver-infant relationship is growing. Numerous reviews have investigated the efficacy and effectiveness of attachment-based interventions (e.g., Bakermans-Kranenberg et al., 2003, 2005; Broberg, 2000; Cornell & Hamrin, 2008; Drozd et al., 2018; Kerr & Cossar, 2014; Letourneau et al., 2015; Mountain et al., 2017; Wright & Edgington, 2016; Wright et al., 2017). Findings have demonstrated improvements in caregiver and infant outcomes through positive impacts on the caregiver-infant relational processes. However, some authors suggested that due to poor study quality in a number of studies, these caregiver-infant outcomes remain somewhat inconclusive (Drozd et al., 2018; Kerr & Cossar, 2014; Wright & Edgington, 2016; Wright et al., 2017, 2023). Furthermore, there is an emerging body of evidence evaluating the effects of video-feedback within attachment-based intervention research. Video-feedback has become a popular and widely used approach within attachment-based interventions (Balldin et al., 2018). Although reviews, including a meta-analysis, have suggested that video-feedback is an effective technique for enhancing caregiver-infant outcomes (Balldin et al., 2018; Fukkink, 2008; O’Hara et al., 2019), many programmes have used a combination of approaches and the extent to which video-feedback was crucial to their overall outcomes is unclear. Nevertheless, this current review aimed to evaluate alternative attachment-based interventions that can complement the extensive evidence-base of video-feedback interventions (see Balldin et al., 2018; Fukkink, 2008; O’Hara et al., 2019; Van Ijzendoorn et al., 2023; Wan et al., 2024).

Health services encourage the use of evidence-based interventions that are accompanied by a manual for training and implementation purposes (Department of Health, 2008). Manuals outline key elements and techniques of interventions, such as session duration and content (Cuijpers et al., 2024). They offer a method of increasing internal validity (Ball et al., 2002) and support professionals in accessing appropriate and standardised intervention training and delivery (Forbat et al., 2015). Consequently, the use of manualisation implies the relatively rapid implementation of an intervention following a thorough training and accreditation procedure, with embedded ongoing supervision.

With the expansion of perinatal and parent-infant mental health services in the United Kingdom (UK), under the National Health Services (NHS) long term plan (LTP; NHS, 2019) and across other countries (e.g., O’Brien et al., 2023), it is imperative to revisit and review the current evidence-base of attachment-based interventions to ensure that interventions with high-quality positive outcomes in the caregiver-infant relationship can be implemented. As no review to date has focussed exclusively on attachment-based interventions supported by manualisation, the current review aimed to explore and evaluate the effectiveness of manualised attachment-based interventions on the relationships of caregivers and infants under two years. To do this, two search stages were conducted to answer the following questions: 1) What manualised attachment-based interventions exist for caregivers and infants from conception to two years? 2) What is the evidence-base of the included interventions in improving caregiver-infant relational outcomes?

## Method

This systematic review was conducted and reported according to the PRISMA framework (Page et al., 2021) and it was registered with PROSPERO (CRD42020206630).

### Stage 1: Identification of Manualised Attachment-Based Interventions

Five databases (CINAHL plus, EMBASE, Medline, PsycINFO and Web of Science) were systematically searched in January 2020 and updated in October 2023 for published, peer-reviewed studies in the English language containing any of the following key terms/concepts within titles and abstracts: “*attachment*”, “*intervention*”, “*parent*”, “*infant*”. University information specialists assisted in identifying terms and Medical Subject Headings (MeSH) were used, when appropriate. The searches included Boolean operators (“OR”, “AND”, “NOT”); “OR” was used within each term/concept and “AND” was used to combine across group terms/concepts for (1) “*attachment*” AND “*intervention*”, (2) “*parent*” AND “*infant*”, then, (3) groups 1 AND 2 were combined. Publication year was not a restriction. A detailed example of the full database search strategy for Stage 1 can be found in Appendix A. To supplement database searches, experts in the perinatal field were consulted, forward and backwards searching was undertaken, and existing reviews were also examined for references.

Stage 1 inclusion criteria were: (1) an attachment-based intervention suitable for pregnant mothers, parents and/or caregivers of infants from conception to two years (because perinatal mental health services in the UK will extend their service provision to this infant age; NHS, 2019), and (2) the intervention was supported by a manual (i.e., written operational guidance instructs the intervenor/therapist on how to deliver or apply the intervention). Drawing on the definitions of attachment-based interventions, the following criteria were operationalised by the research team and adopted for inclusion in the review: (1) the intervention was fundamentally based on the principles of attachment theory (enhancing containment, reciprocity, caregiver sensitivity, attunement, responsiveness, or internal representations of the relationship) and (2) the intervention targeted and aimed to change the quality of the caregiver-infant relationship and/or attachment. Interventions often referred to as ‘parenting’ interventions, based wholly on the principles of social learning theory (e.g., supporting the parent’s ability to manage their child’s behaviour through rewarding/praising positive behaviour, limit setting and applying consistent boundaries for undesirable behaviour; Ryan et al., 2017), were excluded because they usually target groups of children over two years and focus on child behaviour change. Interventions were also excluded if they aimed specifically at caregivers of children with intellectual, neurodevelopmental or physical health disabilities, because there is evidence to suggest the experiences and challenges faced by these parents are distinct from those parents of children without identified significant additional needs (Bourke-Taylor & Jane, 2018). Interventions using video-feedback as the main or core component, as identified by intervention developers, corresponding authors or suggested within the literature that video-feedback was a key or standalone component, were also excluded because they were extensively reviewed elsewhere (see Balldin et al., 2018; Fukkink, 2008; O’Hara et al., 2019; Van Ijzendoorn et al., 2023; Wan et al., 2024). See Appendix B for an overview of the excluded video-based attachment interventions retrieved from the searches.

### Stage 2: Reviewing the Evidence-Base of Identified Attachment-Based Interventions

To review the evidence for each eligible intervention, four relevant databases were systematically searched: CINAHL Plus, EMBASE, PsycINFO and Web of Science. Information specialists, conferred for advice and guidance to optimise the search terms, advised not to use Medline at this stage due to this database returning insufficient results from initial pilot searches. Databases were searched in June 2020 and updated in October 2023, for peer-reviewed studies, published in English only, using the following terms and Boolean operators: “attachment*” OR “*relationship*” OR “*interaction*” OR “*dyad*” OR “*bond*” OR “*sensitivity*” OR “*responsiveness*” OR “*attunement*” OR “*reflexivity*” AND the intervention name OR abbreviation. MeSH terms were again used and the intervention name was searched as a keyword. See Appendix C for an example of the full database search strategy for this stage.

Studies identified in Stage 1, reference lists of included articles and systematic reviews and internet webpages (e.g., Early Intervention Foundation (2017): https://guidebook.eif.org.uk; California Evidence-based Clearinghouse for Child Welfare (2019): https://www.cebc4cw.org/; bibliographic sections of intervention websites) supplemented the database searches. For Stage 2, the review team followed the PICOS framework (Schardt et al., 2007) for assessing eligibility and selecting studies. Table 1 offers further details on the inclusion/exclusion criteria for Stage 2.

**Table 1:** Inclusion and exclusion criteria for Stage 2

	Inclusion	Exclusion
Population	• Participants who are pregnant mothers and/or parents/caregivers of a child aged two years and under	• Studies that include only parents/caregivers of an infant or child with an intellectual, neurodevelopmental, or physical health disability
		• Studies in which more than 50% of the child sample involved are over two years
Intervention	• Interventions identified in Stage 1	
Comparison	• None, an alternative intervention (including another attachment-based intervention), a control group or treatment-as-usual	
Outcome	• Must include a relational measure of the dyad (i.e., a measure of relational functioning, processes, bond or connection between caregiver and infant)	• Post-outcome measures which exceed the infant sample being two years
	• Must be observer-rated, self-report or clinician-rated	• More than 50% of the infant sample are over two years when post outcome measures are taken
Study design	• Quantitative or mixed-method studies	• Case studies and case series designs
	• Within-group or group comparison studies	• Studies using only qualitative methodologies
	• Randomised controlled trials, pilot studies, pre and post-test studies and cohort studies	

### Screening and Data Extraction

Stage 1 and Stage 2 references were imported into bibliographic referencing software (Clarivate Analytics UK Ltd [Version X9], 2020) for screening. The main reviewer (CC) performed the literature searches and screening for both review stages under supervision of the review team (AW & MWW), and with additional support from another reviewer (AR) for the updated search. At Stage 1, all sources were screened based on both the title and abstract and categorised into interventions with and without a clear name. Each intervention was assessed for full eligibility against the inclusion criteria after the following data were extracted: intervention name, aims, age of target infant/child sample, presence of a manual.

Publications were also screened based on both title and abstract at Stage 2. Any studies that could not be excluded from the title and/or abstract were further screened based on their full text. At this stage, an independent reviewer (TP) screened and assessed 10% of identified sources to check for agreement, which was excellent (screening: 98.4%, *k* = 0.88; full-text assessment: 100%, *k* = 1). Any discrepancies were discussed and resolved between the two reviewers. To support screening and data extraction, intervention developers or corresponding authors were contacted via email, up to three times, for any additional or missing information. All extracted data were tabulated for both stages of this review and over 50% of the extracted data were verified by a third-party researcher.

### Quality Assessment and Data Analysis

A methodological quality/risk of bias assessment was performed at Stage 2 only, using the *Quality Assessment Tool for Studies with Diverse Designs* (QATSDD; Sirriyeh et al., 2012). This 16-item-checklist was used because it has good reliability and validity (Fenton et al., 2015) and because diverse study methodologies were noted during pilot searches. Each QATSDD item was rated on a 4-point-scale from “not at all” (0) to “complete” (3). Percentage scores were reported out of a maximum total score of 42, or 48 for mixed-method studies.

Studies scoring over 75% were considered high quality, 50–75% good quality, 25–50% moderate quality and below 25% poor quality, consistent with previous reviews (Gillham & Wittkowski, 2015; Medford et al., 2017). The main reviewer and an independent coder rated all papers. An exact agreement was achieved on 90% of the quality criteria. Any discrepancies were resolved through discussion. As the QATSDD has been critiqued for not addressing important quality aspects of quantitative study designs, including blinding, sequence allocation and randomisation (Fenton et al., 2015). Finally, as recommended by Lucas et al. (2007), findings for Stage 2 were synthesised using a textual narrative synthesis approach (see Barnett-Page & Thomas, 2009, for details).

## Results

**Fig. 1:**
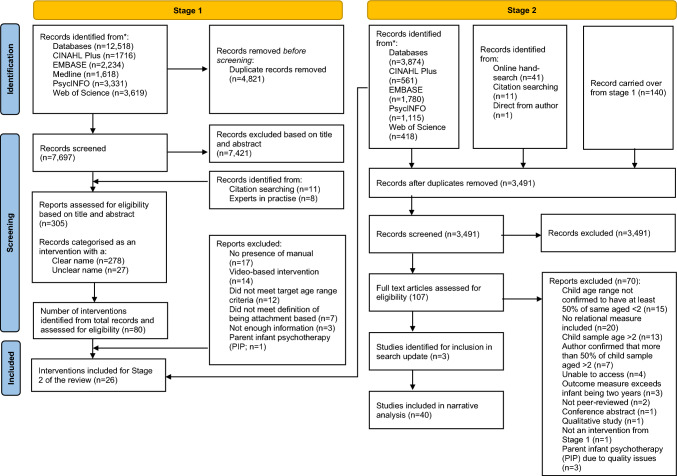
PRISMA flow diagram showing the two stages of this systematic review. *For full lists of exclusions for Stage 1 and Stage 2, please see Appendix D and E

The search process and results are presented in Fig. 1.

### Intervention Characteristics

At Stage 1, 26 manualised attachment-based interventions were identified for inclusion and are summarised in Table 2. The interventions were developed between 1979 and 2019. Most originated in the UK (n = 11) and the United States of America (USA; n = 10). Interventions were universal (n = 8), or developed with specific target groups in mind, including caregiver-infant groups in ‘at risk’ categories (n = 15, i.e., living with adversity or in socioeconomic deprivation, parental mental health difficulties, parental substance misuse, known to child protection services), adoptive/foster parents (n = 2) or adolescent/young mothers (n = 1).

**Table 2:** Summary of the 26 included manualised attachment-based interventions (presented in alphabetical order)

	Developer(s), year, location and website (if applicable)	Aims and format	Target infant age range	Target parent group	Recommended delivery	Recommended number of sessions and duration	Manual availability and access	Author of manual	Details of training (if applicable)	Evidence-base		
										RCT	Group comparison	Other design
1	***Attachment and Biobehavioral Catch-Up (ABC)***											
	Dozier et al. (2002) USA http://www.abcintervention.org	Assist caregiver understanding of infant’s signals and appropriately providing a nurturing and consistent environment in response PE, PL, VOF, IV	Six months – two years	Families experiencing adversity	Caregiver-infant 1:1 CS	Weekly, ten sessions 60 min per session	Available to those who have completed training	Dozier et al. (2002)	Training was available at: http://www.abcintervention.org/training	7	1	1
2	***Baby Bonding***											
	Maskell-Graham (2008) Ireland https://www.bigtoeslittletoesirl.com	Develop a strong relationship before and after birth, so caregivers feel experts in care and play; to help manage the transition into parenthood and build a support network PSS, PE	28 weeks pregnancy – three years	Universal	Caregiver-infant 1:1 and/or group CS	Weekly, eight sessions 60 min per session	Available to purchase	Maskell-Graham (2016)	Training was available at: https://bigtoeslittletoesirl.com/baby-bonding	–	–	–
3	***Circle of Security (COS)***											
	Marvin et al. (2002) USA https://www.circleofsecurityinternational.com	Developing sensitivity by improving caregiver observational skills and responding sensitively to the infant’s needs IV, PE	Birth – five years	High-risk groups (i.e., enrolled with Early Head Start, irritable babies, teens), caregivers, foster parents	Caregiver only group CS	Weekly, eight sessions 90 min per session	Available to those who have completed training	Cooper et al*.* (2000, unpublished manual)	Training was available at: https://www.circleofsecurityinternational.com/trainings/about-trainings	3	1	1
4	***First Play***											
	Courtney (year unknown) USA https://www.firstplaytherapy.com	Enhance caregiver-infant bonding through playful touch and give infants the foundation to make healthy interpersonal relationships ST, PL, PMT	Birth – two years	Universal	Caregiver-infant 1:1 and/or group HV and CS	No information	Available to those who have completed training	No citation available	Training was available at: https://firstplaytherapy.com/firstplay-therapy-training-2-3	–	–	–
5	***Foundations for Attachment***											
	Golding (2017) UK https://www.kimsgolding.co.uk	Increase caregiver understanding of children and infants emotional and behavioural needs; improve trust in relationships, increase skills and social support to promote attachment PE, PSS	Birth – 18 years (groups arranged by similar age ranges)	Adoptive and foster parents	Caregiver only group CS	Weekly, six sessions Three hours per session	Available to purchase	Golding (2017)	Training was available, at: https://kimsgolding.co.uk/resources/programmes/train-the-trainers/	–	–	–
6	***Group Attachment-Based Intervention (GABI)***											
	Murphy et al. (2015) USA	Enhancing caregiver coping, resilience and improving attachment relationships M, PE, VOF	Up to 36 months	Isolated, marginalised families or families who have experienced trauma	Caregiver-infant group CS	Three times a week for 26 weeks Two hours per session	Available upon request or after completion of training	Murphy et al., (2012, unpublished)	Training was available at: https://center-for-attachment.com/training-opportunities-2	1	1	1
7	***Lighthouse MBT Parenting Programme***											
	Byrne et al. (2019) UK http://www.lighthouseparenting.net	Build, enhance and restore caregiver mentalizing to help with their relationships with their infants M, PE, PL	Birth—2 years	Parents who experience complex difficulties	Caregiver only 1:1 and/or group CS	Weekly, 20 sessions Duration unknown	Available upon request	No citation available	Training was available at: http://lighthouseparenting.net/training	–	–	1
8	***Mellow Babies***											
	Puckering et al. (2010) UK https://www.mellowparenting.org	Improve caregiver wellbeing and interactions with their infants PE, M, VOF	6–18 months	Mothers or fathers typically with mental health problems, learning disabilities, forensic issues, drug users, looked after by the local authority (groups arranged by caregiver gender)	Caregiver only 1:1 and caregiver-infant group and/or 1:1 CS	Weekly, 14 sessions Full day per session Additionally, five hours of weekly group sessions for infants and parents	Available to those who have completed training	No citation available	Training was available at: https://www.mellowparenting.org/our-training	1	1	-
9	***Mellow Bumps***											
	The Mellow Team (2012) UK https://www.mellowparenting.org	Decrease stress levels in pregnancy and educate that babies are prepared for social interaction from birth; emphasise the importance of early interaction for brain development PE, M, VOF	Pregnancy (20–30 weeks gestation)	Parents with mental health problems, learning disabilities, forensic issues, drug users, looked after by the local authority	Caregiver only group CS	Weekly, six sessions Two hours per session	Available to those who have completed training	No citation available	Training was available at: https://www.mellowparenting.org/training/	–	–	–
10	***Mellow Parenting***											
	Puckering et al. (1994) UK https://www.mellowparenting.org	Improve relationships by promoting sensitive caregiving to enhance attachment and caregiver mental health PE, M, VOF	Birth – five years	Parents with mental health problems, learning disabilities, forensic issues, drug users, looked after by the local authority	Caregiver only and caregiver-infant group CS	Weekly, 14 sessions Full day per session	Available to those who have completed training	No citation available	Training was available at: https://www.mellowparenting.org/training/	–	–	–
11	***Minding the Baby (MTB)***											
	Slade et al. (2005) USA Link was: https://www.learning.nspcc.org.uk/services-children-families/minding-the-baby	Develop caregiving reflective capacities, to recognise and respond to infant’s feelings and needs; develop a positive relationship and secure bond with the infant M, VOF	Birth—2 years	First-time mothers who require benefits, aged 25 and under	Caregiver-infant 1:1 HV	Weekly through pregnancy until the infant’s first year then fortnightly until the infant turns two 60 min per session	Available to those who have completed training	Slade et al. (2004c) Slade et al. (2018)	Training was available at: www.mtb.yale.edu	2	–	–
12	***Mom Power (MP)***											
	Muzik et al. (2011) USA https://www.zerotothrive.org	Increase attachment through enhancing caregiving skills and self-care skills PE	Birth—6 years	Mothers living with adversity	Caregiver only and caregiver-infant group CS	Ten weekly group sessions and three individual sessions Three hours per session	Available to purchase	No citation available	Training was available twice a year or upon request	–	–	–
13	***Mothers and Babies Course (MB)***											
	Muñoz et al. (2007) USA https://www.mothersandbabiesprogram.org	To help pregnant women cope with the stress of parenting and manage their mood using cognitive behavioural therapy skills PE, PSS	Pregnancy	Universal	Caregiver only group or 1:1 CS or HV	Weekly, six–nine sessions Optional booster sessions at one, three, six and 12 months postpartum 20–25 min per session (1:1) Two hours per session (group)	Available via Internet weblink	No citation available	Training was available at: https://www.mothersandbabiesprogram.org/providers/mb-training	2	–	–
14	***Mothers and Toddlers Program (MTP)***											
	Suchman et al. (2008) USA	To shift caregivers representational balance and capacity for reflective functioning and increase caregiver capacity for sensitivity and responsiveness PE, M	Birth – 36 months	Substance abusing mothers	Caregiver only 1:1 CS	Weekly, 12 sessions 60 min per session	No information	No citation available	No information	–	–	–
15	***New Beginnings***											
	Baradon et al. (2008) UK	Enhance caregiver attunement to the infant and prepare caregivers for the separation that will occur M, PE	Birth – 12 months	Prison-based mothers, homeless mothers, children on the child protection register	Caregiver-infant group Prison MBU/CS	Twice a week, eight sessions Two hours per session	Available to those who have completed training	Baradon (2009, unpublished)	Training can be commissioned	2	–	–
16	***Nurturing Attachments Group***											
	Golding (2007, unpublished) UK https://www.kimsgolding.co.uk	Enhance sensitive caregiving to increase feelings of safety and attachment M, PE	Birth – 18 years (groups arranged to similar age ranges)	Adoptive parents	Caregiver only group CS	Weekly, three modules of six sessions (18 sessions total) Three hours per session	Available to purchase	Golding (2014)	Training was available at: https://www.mellowparenting.org/training/	–	–	–
18	***Right from the Start (RFTS)***											
	Niccols (2008) UK	Enhance caregiver skills in reading infant cues and responding sensitively PE	One – 24 months	Mothers who have infants at risk of attachment difficulties and/or universal	Caregiver only 1:1 and/or group HV and CS	Weekly, eight sessions Two hours per session	Available to purchase	Niccols et al. (1999)	No information	1	–	–
19	***Secure Attachment Family Education (SAFE)***											
	Brisch (2010, 2017—unpublished) Germany	Prevent the transmission of unresolved trauma from caregivers to an infant IV, PSS, PE, VOF	Pregnancy – 12 months	All parents to be	Caregiver only 1:1 and caregiver-infant group CS	Four group sessions and one individual session held before birth, six group sessions and two individual sessions held after birth Seven hours per session	No information	No citation available	Training was available at: https://www.khbrisch.de/en/downloads/flyer/flyer-for-the-safe-mentor-training-english/?showId=166	1	–	–
20	***Secure Attachment Promotion Program***											
	Santelices et al. (2011) South America	Promote caregiver sensitivity, change mental representations, and promote the development of an infant’s secure and healthy bond PE, M, IO	Birth – two years	Middle and lower-class women who require prenatal medical care	Caregiver-infant 1:1 and caregiver only group CS	Weekly, six sessions whilst pregnant, sessions offered across four occasions during the infant’s first year 60 min per session	No information	No citation available	No information	1	–	–
21	***Strengthening Relationships Towards Secure Attachment***											
	Leigh et al. (2013) South America	Promote attachment security in caregiver-infant dyads detected in primary health care PE, BS	Six – 12 months	Universal	Caregiver-infant 1:1 and/or group and caregiver only group CS	Weekly, six sessions whilst pregnant, sessions offered across four occasions during the infant’s first year 60 min per session	No information	No citation available	Training was provided to healthcare workers for the research. However, this is not readily available	–	–	1
22	***The Solihull Approach***											
	Douglas and Ginty (2001) UK https://www.solihullapproachparenting.com	Enhance containment, reciprocity, attunement and behaviour management; encourage caregiver sensitivity through the role of communication PE, M	Pregnancy – 18 years	Universal	Caregiver only group HV and CS	Weekly, ten sessions Two hours per session	Various manuals available to purchase	Solihull NHS Primary Care Trust. (2006) Douglas (2006, unpublished manual)	Training was available at: https://solihullapproachparenting.com/trainings	–	–	2
23	***Theraplay***											
	Jernberg (1979) USA https://www.theraplay.org	Enhance infant attachment, self-esteem, trust in others and engagement through play, games, and other bond strengthening activities PL, PE	Birth – 18 years	Universal	Caregiver-infant 1:1 and/or group CS	Weekly, 19–28 sessions, with four follow-up sessions over the next year 30–45 min per session	Available to purchase	Booth and Jernberg (2009)	Training was available at: https://theraplay.org/training/training-programs	–	–	–
24	***Thula Sana***											
	Cooper et al. (2002) UK	Promote caregiver sensitivity and responsive interactions between a mother and her infant PE, IO	Pregnancy – six months	Socioeconomically deprived mothers	Caregiver only and caregiver-infant 1:1 HV	Weekly, 16 sessions, twice antenatally, weekly for first eight weeks postpartum, fortnightly for further two months, monthly for two months Duration unknown	Available via internet weblink	World Health Organisation (2019)	Training is no longer being provided	1	1	–
25	***UCLA Family Development Project***											
	Heinicke et al. (1999) USA	Enhance the ability of family members to care for themselves, each other, cope with life stressors and build a bond with their infant PSS, M, PE	Late pregnancy – four years	Mothers who are at risk of ‘inadequate parenting’	Caregiver only 1:1 HV	Weekly until one year old, fortnightly until two years old, then regular telephone contact until aged four 60 min per session	No information	Heinicke (2000)	No information	1	1	1
26	***Watch Me Play!***											
	Wakelyn (2011) UK https://tavistockandportman.nhs.uk/watch-me-play	Encourages child-led play, direct attention from caregivers and discussions with the child about play PL, PE	Birth—5 years	Those with concerns about caregiver-parent relationships or parental mental health difficulties, including foster caregivers	Caregiver-infant 1:1 HV	Weekly, six sessions Option for extra 5–6 sessions if necessary	Available via Internet weblink	Wakelyn and Katz (2020)	Training was available at: https://tavistockandportman.nhs.uk/training/cpd-courses/introduction-watch-me-play/	–	–	–
27	***Watch, Wait and Wonder (WWW)***											
	Cohen et al. (1999) Canada https://www.watchwaitandwonder.com	Enhance caregiver sensitivity and responsiveness, the infant’s sense of self and self-efficacy, emotion regulation, and the attachment PL, PE, M	Ten – 30 months	Universal	Caregiver-infant 1:1 CS	Weekly, 15 sessions 60 min per session	Available to purchase	Muir et al. (1999)	Training was available at: https://watchwaitandwonder.com	–	2	–

B = other behavioural strategies; CS = community setting; HV = home visitation; IO = infant observation; IV = instructional video; M = mentalisation; MBU = mother and baby unit; PE = psychoeducation; PL = play; PMT = parent massage training; PSS = psychosocial support; ST = storytelling; VOF = video-observation feedback

Interventions were delivered on an individual basis (caregiver only and/or caregiver- infant dyad; n = 7), in group settings (caregiver only and/or caregiver-infant dyad; n = 6) or using combinations of individual and group working (n = 13). Most interventions were conducted weekly and ranged between six to over 70 sessions, with some decreasing in frequency over time. Sessions varied between 20 min to a full day. Settings included a community location (e.g., outpatient clinic or children’s centre; n = 17), home visitation (n = 1), or a combination of both (n = 4). The intervention approaches included assortments of psychoeducation (n = 22), mentalisation (n = 14), some video-observation feedback (n = 8), play (n = 7), instructional videos (n = 4), parent psychosocial support (n = 5), infant observation (n = 2), behavioural strategies (n = 1), parent massage training (n = 1) and storytelling (n = 1). Two interventions (*Mellow Parenting* and *Right from the Start*) followed a single approach, whilst the remainder used a multi-modal approach (n = 24). Of the included interventions incorporating a multi-model approach, seven interventions were found to use an element of video-feedback; however, this was not key to the intervention delivery and the approach was less salient than the other featured components.

### Evidence-Base for Studies with Relational Outcome Measure(s)

In total, empirical evidence or studies in which a relational outcome was evaluated was found for 16 interventions: *Attachment and Biobehavioral Catch-Up* (ABC^1^; n = 9), *Circle of Security* (COS; n = 5), *Group Attachment-Based Intervention* (GABI; n = 3), *Lighthouse MBT Parenting Programme* (n = 1), *Mellow Babies* (n = 2), *Minding the Baby* (MTB; n = 2), *Mothers and Babies Course* (MB; n = 2), *New Beginnings* (n = 2), *Right from the Start* (RFTS; n = 1), *Secure Attachment Family Education* (SAFE; n = 1), *Secure Attachment Promotion Program* (n = 1), *Strengthening Relationships Towards Secure Attachment* (n = 1), *The Solihull Approach* for pregnancy and infancy only (n = 2), *Thula Sana* (n = 3), *UCLA Family Development Project* (n = 3) and *Watch, Wait and Wonder* (WWW; n = 2).

The following interventions did not meet this review’s eligibility criteria for evidence to be included: *Baby Bonding*, *First Play, Foundations for Attachment, Mellow Bumps, Mellow Parenting, Mom Power*, *Mothers and Toddlers Program*, *Nurturing Attachments Group*, *Theraplay* and *Watch Me Play!*. However, developers, who were emailed, indicated that there were studies that were either being conducted or awaiting imminent publication for four interventions: *Baby Bonding, COS, Lighthouse Parenting MBT Programme, Strengthening Relationships Towards Secure Attachment* and *Watch Me Play!*. Therefore, future evidence is to be expected and considered.

Study designs were Randomised Controlled Trials (RCT; n = 23, including 18 full RCTs and five pilot RCTs), one non-RCT, independent groups comparison studies (n = 5) and within-group designs (n = 11). Seven interventions had RCT evidence published, one intervention had independent group comparison evidence only and three interventions had studies using within-groups design only. Six interventions had a combination of evidence. *ABC* had the most studies eligible for inclusion in this review (n = 9) and the most RCT evidence (n = 7), see Table 2 for details.

### Methodological Quality of Studies

The methodological quality of studies ranged from 33.3% (*The Solihull Approach*: Harris-Waller et al., 2019) to 88.3% (*MTB*: Slade et al., 2020). Most studies scored within the good quality range (n = 27) and no studies were found to be of poor quality (see Table 3). Interventions that were considered to have high quality rated studies included: *ABC* (n = 1; Perrone et al., 2020), *COS* (n = 3; Maxwell et al., 2020; Cassidy et al., 2011; Ramsauer et al., 2019), *GABI* (n = 1; Steele et al., 2019), *MB* (n = 1; McFarlane et al., 2017), *MTB* (n = 2; Slade et al., 2020; Sadler et al., 2013) and *Thula Sana* (n = 2; Valades et al., 2021; Cooper et al., 2009).

**Table 3:**
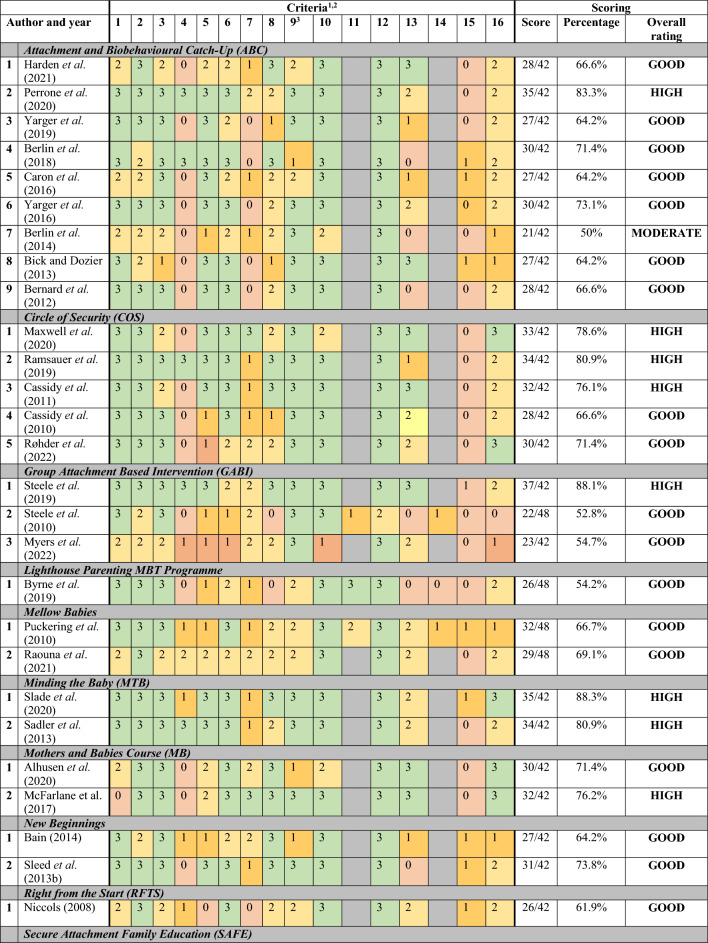

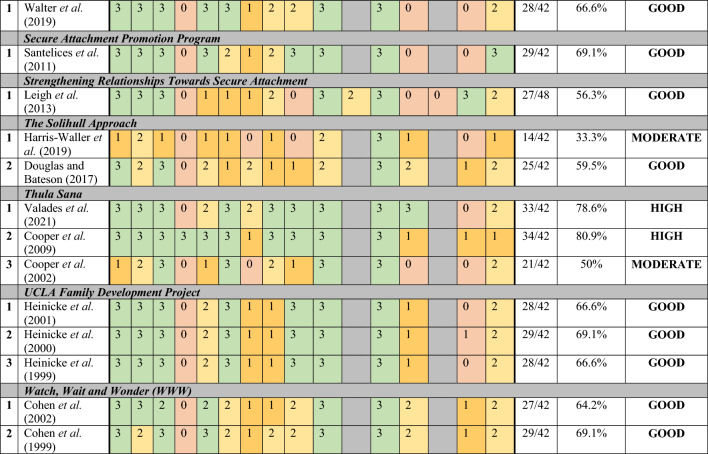
Methodological quality of the included studies

^a^Some aspects of different study designs were considered independently

^b^QATSDD Criteria: (1) explicit theoretical framework; (2) clear aims/objectives; (3) description of research setting; (4) sample size considered for analysis (5) representative sample size of target population; (6) CLEAR procedure for data collection; (7) rationale for choice of data collection tool(s); (8) detailed recruitment data; (9) assessment of reliability^a^; (10) fit between research question and method of data collection (quantitative); (11) fit between research question and method of data collection (qualitative); (12) fit between research question and method of analysis; (13) good justification for analytical method selected; (14) assessment of reliability (Qualitative); (15) Evidence of user involvement in design of study; (16) Strengths and limitations. QATSDD scoring: 0, not at all, 1, very slightly, 2, moderately, 3, complete

^c^Inter-rater reliability assessment must have been completed

Reasons for low ratings included studies with no power analysis (criterion 4), limited rationale for the choice of data collection tools (criterion 7) and analytical method (criterion 13) and limited evidence of inclusion of service users in the study design (criterion 15). Following observation of the year of publication, studies from six interventions (*COS, GABI, MTB, New Beginnings, The Solihull Approach* and *Thula Sana*) demonstrated study methodological quality improvement over time. Additionally, of the studies using controlled trials or group comparison designs (n = 30), 25 studies (62.5%) reported randomly allocating participants to intervention groups, three reported using quasi-random allocation (7.5%) and one study used a non-random allocation. The method of allocation to intervention group was described in 12 of those studies (30%). Methods of blinding were reported in 30 studies of the total included (75%).

### Study Characteristics

Table 4 offers a summary of included study characteristics and relational findings (additional study characteristics can be found in Appendix F). Studies were conducted between 1999 and 2022 in the USA (n = 21), UK (n = 6), Germany (n = 2), Canada (n = 3), South Africa (n = 3), Chile (n = 2), Australia (n = 1), Denmark (n = 1), and El Salvador (n = 1). Sample sizes ranged from 11 (*Strengthening Relationships Towards Secure Attachment*: Leigh et al., 2013) to 449 (*Thula Sana*: Cooper et al., 2009) participants. Samples mostly comprised of mother-infant dyads (n = 33, 82.5%, including pregnant mothers). Thirty-one studies (77.5%) involved samples from specific subgroups or ‘at risk’ categories (i.e., homeless, known to child protection services, parental mental health difficulties) and 25 studies (62.5%) included samples of mother-infant dyads. Fourteen studies (35%) fulfilled the infant age criteria, although had a percentage of the infant sample aged over two years.

**Table 4:** Overview of study findings for the 16 interventions reporting on relational outcomes

	Authors, location and quality	Study design and method^a^	Sample and intervention vs. control(s) details	Additional sample characteristics and percentage of infant sample over 2 years?	Caregiver-infant relational measures and variables (including timepoint of administration^b^)	Reported caregiver-infant relational main findings (including p-value if reported)^c^
	***Attachment and Biobehavioral Catch-up (ABC)***					
1	Harden et al. (2021) USA Good 66.6%	RCT *Multi-timepoint:* T1: pre-intervention T2: post-intervention	Mother-infant dyads ABC (n = 104) vs. Early Head Start & “Book-of-the-Week” (n = 104)	Predominantly Latino, low-income families No	QRCI: dyadic mutuality (emotional synchrony, shared experience reciprocal play, communication, mutual interest, warmth and enjoyment of each other)	Post-intervention dyadic mutuality, *p* < 0.05*, *d* = 0.27 Dyadic mutuality was moderated by maternal risk, *p* = 0.003*
2	Perrone et al. (2020) USA High 83.3%	RCT *Multi-timepoint:* T1: pre-intervention T2: post-intervention	Mother-infant dyads ABC (n = 100) vs. waitlist (n = 100)	Living in poverty and involved in child welfare services No	NICHD ORCE: sensitivity, intrusiveness and positive regard	Post-intervention sensitivity, *p* = 0.04*, *d* = 0.21 No significant group differences to post-intervention intrusiveness or positive regard
3	Yarger et al. (2019) USA Good 64.2%	RCT *Single timepoint:* post-intervention (following completion)	Mother-infant dyads ABC (n = 50) vs. Developmental Education for Families (n = 55)	Known to child protective services Yes	SSP AMBIANCE: parental withdrawal, disruptive parenting, communication errors, role/boundary confusion, fearful/disorientated parenting and intrusive/negative parenting	Post-intervention attachment disorganisation (44% vs. 39%), *p* = 0.16 Post-intervention parental withdrawal, *p* = 0.03*, *d* = − 0.42 No significant group differences to post-intervention affective communication errors, *p* = 0.33; role/boundary confusion, *p* = 0.74; fearful/disoriented parenting, *p* = 0.52; intrusive/negative parenting, *p* = 0.60
4	Berlin et al. (2018) USA Good 71.4%	RCT *Multi-timepoint:* T1: pre-intervention T2: post-intervention (within one month of completion)	Mother-infant dyads ABC (n = 104) vs. Early Head Start and ‘Book of the week’ (n = 104)	Latino and receiving Early Head Start home-based services No	QRCI: maternal sensitivity, intrusiveness and positive regard AAA^(T1)^ ECR^(T1)^	Post-intervention sensitivity, *p* ≤ 0.0001*, *d* = 0.27; decreased intrusiveness, *p* ≤ 0.001*, *d* = 0.77; positive regard, *p* = 0.01*, *d* = 0.23 Maternal sensitivity by attachment status, secure, *p* = 0.001*, *d* = 0.49; anxious, *p* = 0.038*, *d* = 0.30; avoidant, *p* = 0.008*, *d* = 0.38
5	Caron et al. (2016) USA Good 64.2%	Within-group *Multi-timepoint:* T1: pre-intervention T2: post-intervention (following final session)	Parent-infant dyads ABC (= 78) vs. benchmarks found in RCTs (Bernard et al., 2015; Dozier & the infant-caregiver project, 2015)	N/A Yes	NICHD ORCE: caregiver following the lead, delight and intrusiveness	Post-intervention results were comparable to RCTs, following the lead increased by 1 point, *p* = 0.002*, *d* = 0.89; delight increased by 0.4 points, *p* = 0.047*, *d* = 0.41; intrusiveness decreased by 1.3 points, *p* ≤ 0.001*, *d* = − 1.21
6	Yarger et al. (2016) USA Good 73.1%	RCT *Multi-timepoint:* T1: pre-intervention T2: post-intervention (during follow up visit)	Mother-infant dyads ABC (n = 13) vs. Developmental Education for Families (n = 11)	N/A Yes	NICHD ORCE: sensitivity and intrusiveness	Post-intervention sensitivity, *p* = 0.04*, *d* = 0.70; decreased intrusiveness, *p* = 0.02*, *d* = − 0.81 Mothers showed steeper rates of change to sensitivity and intrusiveness during the first half of the treatment vs. second half, *p* = 0.03*
7	Berlin et al. (2014) USA Moderate 50%	Pilot RCT *Multi-timepoint:* T1: pre-intervention T2: post-intervention (two weeks following completion)	Mother-infant dyads ABC (n = 11) vs. ‘Book of the week’ (n = 10)	Receiving residential substance abuse treatment No	MBQS^(T2)^	No significant group difference to post-intervention sensitivity, *p* = 0.096
8	Bick and Dozier (2013) USA Good 64.2%	Independent groups comparison *Multi-timepoint:* T1: pre-intervention T2: one month following completion T3: infant at 12 months T4: infant at 24 months	Foster mother-infant dyads ABC (n = 44) vs. Developmental Education for Families (n = 52)	N/A No	Observer-rated play interaction coded via five-point Likert scale for sensitivity	24 months sensitivity, *p* = 0.05*
9	Bernard et al. (2012) USA Good 66.6%	RCT *Multi-timepoint:* T1: pre-intervention T2: post-intervention (one month following completion)	Mother-infant dyads ABC (n = 60) vs. Developmental Education for Families (n = 60)	Receiving residential substance abuse treatment Yes	SSP	Post-intervention disorganised attachment, *p* ≤ 0.01*,* d* = 0.52 Post-intervention secure attachment, *p* ≤ 0.05*, *d* = 0.38
	***Circle of Security (COS)***					
1	Maxwell et al. (2020) Australia High 78.6%	Non-RCT *Multi-timepoint:* T1: pre-intervention T2: post-intervention (following final session)	Parents COS (n = 201) vs. waitlist (n = 55)	Self-identified parenting challenges (mainly depression) Yes	Composite Caregiving Questionnaire (designed for the study): caregiver mentalising, self-efficacy (specifically empathy, expressing affection and caregiving helplessness) and perceptions of infant (hostility and difficultness)	Post-intervention caregiver mentalising, *p* = 0.001*,* d* = 0.07; empathy, *p* = 0.001*, *d* = 0.06; expressing affection, *p* = 0.034*, *d* = 0.03, decreased helplessness, *p* = 0.016*, *d* = 0.08; less hostile perceptions of infant, *p* = 0.001*, *d* = 0.06 No significant group difference to infant difficultness perceptions Post-intervention relational differences were associated with depression symptoms, *p* ≤ 0.001*
2	Ramsauer et al. (2019) Germany High 80.9%	RCT *Multi-timepoint:* T1: pre-intervention T2: infant at 16–18 months	Mother-infant dyads COS-intensive intervention (n = 36) vs. treatment as usual (n = 36)	N/A No	SSP^(T2)^ Mini-MBQS AAI^(T1)^	No significant group differences to attachment security, *p* = 0.64, or sensitivity, *p* = 0.36 Mothers reporting higher depressive symptoms showed lower sensitivity, *p* = 0.05* COS group mothers who had an unresolved attachment (22.6%) showed more change in sensitivity compared to those without, *p* = 0.12
3	Cassidy et al. (2011) USA High 76.1%	RCT *Multi-timepoint:* T1: pre-intervention T2: infant at 12 months	Mother-irritable infant dyads COS-intensive intervention (n = 85) vs. three psychoeducational sessions (n = 87)	Irritable infants and their economically stressed mothers No	SSP^(T2)^ ECR	12 months secure attachment vs. control, 60% vs. 50% Highly irritable infants of secure mothers had higher probability of being secure with intervention vs. control: 97% vs. 59% Highly irritable infants of fearful mothers had higher probability of being secure with intervention vs. control: 69% vs. 69% Highly irritable infants of dismissive mothers had higher probability of being secure with intervention vs. control: 96% vs. 45% Highly irritable infants had a greater likelihood of being securely attached than moderately irritable infants with intervention vs. control: 89% vs. 63%
4	Cassidy et al. (2010) USA Good 66.6%	Within-group *Multi-timepoint:* T1: pre-intervention T2: infant at 12 months	Mother-infant dyads COS Perinatal Protocol (n = 20) outcomes compared to other meta-analytic data (van Ijzendoorn et al., 1999). Maternal sensitivity was compared to another study running at the same time (Cassidy et al., 2011)	Participating in a 15-month jail diversion program with a history of substance abuse No	Video-recorded play assessment and unstructured snack time coded using a four-point Likert scale for maternal sensitivity^(T2)^ SSP^(T2)^ ECR^(T1)^ PARQ^(T1)^: Warmth/acceptance, hostility/rejection subscales	12 months secure attachment in comparison to depressed parents, *p* = 0.05*; low socio-economic parents, *p* = 0.05*; substance-abusing mothers, *p* = 0.0001*; maltreated infants, *p* = 0.0001* 12 months disorganised attachment in comparison to abusing mothers, *p* = 0.05*; maltreating mothers, *p* = 0.05*; no significant difference with depressed or low socio-economic samples No significant group difference to maternal sensitivity
3	Røhder et al. (2022) Denmark Good 71.4%	RCT *Multi-timepoint;* T1: study inclusion (3–5 months of pregnancy) T2: infant is 8 weeks old T3: post intervention (child is 9 months old)	Mother-infant dyads (n = 78)	Pregnant women with psychosocial vulnerabilities No	CIB: maternal sensitivity and intrusiveness, infant involvement and withdrawal, dyadic reciprocity, and dyadic negative states ASQ-SE: self-regulation, compliance, communication, adaptive functioning, autonomy, emotions, and interaction with other people	No significant difference found for maternal sensitivity, child socio-emotional development, parental reflective functioning, maternal depressive symptoms, or parental wellbeing
	***Group Attachment-Based Intervention (GABI)***					
1	Steele et al. (2019) USA High 88.1%	RCT *Multi-timepoint:* T1: pre-intervention T2: post-intervention	Mother-infant dyads GABI (n = 43) vs. Systematic Training for Effective Parenting (n = 35)	Serious concerns about the mother’s ability to effectively meet their infant’s emotional needs Yes	CIB: maternal hostility, dyadic constriction, maternal supportive presence and dyadic reciprocity	Post-intervention maternal hostility, *p* = 0.05*, *d* = 0.06; dyadic constriction, *p* ≤ 0.001*, *d* = 0.16; maternal supportive presence, p ≤ 0.01*, *d* = 0.12; dyadic reciprocity, *p* ≤ 0.001*, *d* = 0.19
2	Steele et al. (2010) USA Good 52.8%	Within-group *Single timepoint:* post-intervention	Mother-infant dyads (n = 27)	Serious concerns about the mother’s ability to effectively meet their infant’s emotional needs Yes	SSP AAI	Post-intervention secure attachment, 54% Post-intervention disorganised attachment, 45% 54% of mothers were rated as having an unresolved attachment concerning past trauma
3	Myers et al. (2022) USA Good 57.7%	RCT *Multi-timepoint:* T1: pre-intervention T2: post-intervention T3: six month follow up	Mother-infant dyads (n = 20)	N/A No	CIB AAI RF	Post treatment msternsl praising, *p* ≤ 0.05 Child positive affect corelated with maternal RF,* p* ≤ 0.05 and child alertness* p* < 0.01*
	***Lighthouse MBT Parenting Programme***					
1	Byrne et al. (2019) UK Good 54.2%	Within-group *Multi-timepoint:* T1: pre-intervention T2: post-intervention	Parents (n = 16)	Identified as at risk of disorganised attachment (demonstrated difficulties/fleeting knowledge of their difficult relationship with their infant at assessment) No	NICHD ORCE: sensitivity, non-distress and intrusiveness PDI	Post-intervention sensitivity, *p* = 0.045* No significant difference in parent mentalising capacity, *p* = 0.77; with suggested trends in increased parent reflective function Post-intervention trends suggested in how parents perceived their infant and how positive they felt interactions were with their infant
	***Mellow Babies***					
1	Puckering et al. (2010) UK Good 66.7%	RCT *Multi-timepoint:* T1: pre-intervention T2: post-intervention (four months following completion)	Mother-infant dyads Mellow Babies (n = 11) vs. waitlist (n = 6)	N/A No	Mellow Parenting Observation Coding Scheme: positive interaction (anticipation of the infant’s need, responsiveness, autonomy, cooperation) and negative interaction (distress, control and conflict)	Post-intervention in overall positive interaction, *p* = 0.015*, *d* = 3.12; decrease in overall negative interaction, *p* = 0.07*, *d* = − 1.95
2	Raouna et al. (2021)	Pre-post intervention design	Parent-infant dyads 10 Mellow Mums groups (70 mother-baby dyads) 5 Mellow Dads groups (21 father-baby dyads	At risk parents referred to MP via health visitors No	KPCS^(T1, T2)^: parenting confidence, perceived efficacy of parenting	No significant changes in KPCS (*p* = 0.201)
	***Minding The Baby (MTB)***					
1	Slade et al. (2020) USA High 88.3%	RCT *Multi-timepoint:* T1: prenatally T2: infant at four months T3: infant at 12 months T4: infant at 24 months	Pregnant mothers MTB (n = 77) vs. treatment as usual (n = 79)	Attending prenatal care sessions at community health centres No	SSP^(T3)^ AMBIANCE^(T2)^: affective communication PI^(T1)^ PDI-Revised^(T4)^	12 months secure attachment, *p* = 0.01* No significant group difference to affective communication at four months 24 months reflective function, *p* = 0.04*
2	Sadler et al. (2013) USA High 80.9%	Pilot RCT *Multi-timepoint:* T1: prenatally T2: infant at four months T3: infant at 12 months T4: infant at 24 months	Pregnant mothers MTB (n = 60) vs. treatment as usual (n = 45)	Attending prenatal care sessions at community health centres No	SSP^(T3)^ AMBIANCE^(T2)^: affective communication PI^(T1)^ PDI-Revised^(T4)^	12 months secure attachment, *p* = 0.028*; percentage of infants in the intervention group classified as disorganised vs. control group, *p* = 0.049* Four-month affective communication, *p* = 0.05* 24 months reflective function improved significantly across both groups; intervention mothers’ improvement in mentalisation, *p* = 0.016*
	***Mothers and Babies Course (MB)***					
1	Alhusen et al. (2020) USA Good 71.4%	Pilot RCT *Multi-timepoint:* T1: prenatally (9–12 weeks gestation) T2: prenatally (36 weeks gestation) T3: 12 weeks postpartum	Pregnant mothers MB (n = 30) vs. treatment as usual (n = 30)	Exhibiting moderate to severe depression symptoms No	MFAS^(T1, T2)^ NCAST-feeding^(T3)^: maternal sensitivity during feeding	MB mothers had higher increase in attachment scores vs. control: 12.6 vs. 4.6 MB mothers suggested more favourable levels of maternal sensitivity during feeding
	McFarlane et al. (2017) USA High 76.2%	RCT *Multi-timepoint:* T1: pre-intervention T2: post-interventionT3: six months follow up	Mother-infant dyads MB plus home visitation (n = 49) vs. treatment as usual (n = 46)	Identified as having weaker parenting skills Yes	KIPS^(T1, T3)^: sensitivity of responses, physical interaction, involvement in activities, reasonable expectations and encouragement	Six-month follow up sensitivity of responses, *p* < 0.04*, *d* = 0.50 and involvement in activities, *p* < 0.07, *d* = 0.52 No significant group difference to physical interaction, reasonable expectations and encouragement
	***New Beginnings***					
1	Bain (2014) South Africa Good 64.2%	RCT *Multi-timepoint:* T1: pre-intervention T2: post-intervention (two weeks following completion)	Mother-infant dyads New Beginnings across two homeless shelters (n = 10) vs. comparator homeless shelter (offered more long-term stay) received a different intervention (unnamed) which was delayed for ethical reasons (n = 6)	Mothers with histories of loss and abandonment residing in three homeless shelters from two months to two years Yes	EAS: mother’s ability to structure interactions with infant and infant responsiveness PDI	Post-intervention ability to structure interactions, *p* = 0.015*; mothers who reported that they felt more worthless were less able to structure interactions with their infant, *p* = 0.03* No significant group difference to infant’s responsiveness towards mother 31% of mothers in the intervention reflective function scores increased by 1–2 points
2	Sleed et al. (2013) UK Good 73.8%	RCT *Multi-timepoint:* T1: pre-intervention T2: post-intervention T3: two months follow up	Mother-infant dyads New beginnings (n = 88) vs. MBU prison intervention (n = 75)—although less at follow-up points due to being released or moved to a different prison	Residing in prison mother and baby units No	CIB: dyadic attunement, parent positive engagement and infant involvement PDI^(T1, T4)^ MORS	Two months follow-up increased dyadic attunement, *p* = 0.002*, *d* = 0.102; parent positive engagement, *p* = 0.002*,* d* = 0.22 Two months follow-up warmth perceptions for both intervention groups, *p* = 0.002*, *d* = 0.17 No significant group difference to invasive perceptions Post-intervention reflective function, *p* = 0.0002*,* d* = 0.55; associated with higher infant involvement, *p* = 0.05*
	***Right From The Start (RFTS)***					
1	Niccols (2008) Canada Good 61.9%	RCT *Multi-timepoint:* T1: pre-intervention T2: post-intervention T3: six months follow up	Mothers RFTS (n = 48) vs. treatment as usual (n = 28)	N/A No	AQS MBQS HOME: sensitivity	Six months follow-up secure attachment, *p* = 0.55 Six months follow-up sensitivity, *p* = . 34 Follow-up maternal sensitivity scores related to infant attachment, *p* = 0.05*; which was correlated to a trend in change over long-term infant attachment
	***Secure Attachment Family Education (SAFE)***					
1	Walter et al. (2019) Germany Good 69.1%	RCT *Multi-timepoint:* T1: following first session T2: following final session	Mothers and fathers SAFE (mothers n = 89; fathers n = 83) vs. parallel intervention (mothers n = 80; fathers n = 72)	N/A No	SSP^(T2)^	Infants were more likely to be securely attached to their fathers post-intervention, *p* = 0.049* No significant group difference of infant secure attachment to mothers, *p* = 0.468 Post-intervention secure vs insecure attachment to mother and father, 64.7% vs 8.8%
	***Secure Attachment Promotion Program***					
1	Santelices et al. (2011) Chile Good 66.6%	Pilot RCT *Single timepoint:* Post-intervention	Pregnant mothers Secure Attachment Promotion Program (n = 43) vs. educational talk (n = 29)	From lower and middle-class socio-economic levels who sought prenatal care at medical centres No	SSP	Post-intervention secure attachment vs. control, 72% vs 55% Intervention group attachment status, 65.2% secure attachment, 26.4% avoidant attachment, 8.4% ambivalent attachment
	***Strengthening Relationships Towards Secure Attachment***					
1	Leigh et al. (2013) Chile Good 56.3%	Within-group *Multi-timepoint:* T1: pre-intervention T2: post-intervention (four months following completion)	Mother-infant dyads (n = 11)	Identified as being at risk of having non-secure styles of attachment No	MCS	Post-intervention attachment status, 56% moved from insecure to secure attachment Those classified as secure attachment style at the start of the intervention maintained at post-intervention, *p* = 0.125
	***The Solihull Approach***					
1	Harris-Waller et al. (2019) UK Moderate 33.3%	Within-group *Multi-timepoint:* T1: pre-intervention T2: post-intervention (immediately following final session)	Foster carers (n = 56)	N/A Yes	CEFR	No significant group difference to expression of feelings in relationships
2	Douglas and Bateson (2017) UK Good 59.3%	Within-group *Multi-timepoint:* T1: following first session T2: following final session	Mothers and fathers (n = 60)	N/A Yes	MPAAS	Post-intervention increase in attachment for mothers, *p* = 0.001*, *d* = 0.30 and fathers *p* = 0.001*, *d* = 0.51
	***Thula Sana***					
1	Valades et al. (2021) El Salvador High 78.6%	Pilot RCT *Multi-timepoint:* T1: third trimester of pregnancy T2: three months postpartum T3: six months postpartum	Mother-infant dyads Thula Sana (n = 31) vs. no treatment (n = 30)	14–19-year-old first time mothers screened as having symptoms of maternal depression No	Video-recorded face to face play sessions coded using GRS for maternal sensitivity and intrusiveness and infant regulated behaviours (positive vocalisations, positive communicative expressions, direct protest and non-social gaze) and infant dysregulated behaviours (negative vocalisations)^(T3)^	Six months postpartum maternal sensitivity, *p* = 0.015*, *d* = 0.74; decreased intrusiveness, *p* = 0.198, *d* = 0.42 Six months infant positive vocalisations, *p* = 0.29*, *d* = 0.21; positive communicative expressions, *p* = 0.155, *d* = 0.09; direct protest, *p* ≤ 0.001*, *d* = 0.26; non-social gaze, *p* ≤ 0.001*, *d* = 0.38; negative vocalisations, *p* ≤ 0.001*, *d* = 1.6 Thula Sana group infants showed more social regulation strategies and goal-directed non-social behaviours vs. control group, *p* = 0.001*
2	Cooper et al. (2009) South Africa High 80.9%	RCT *Multi-timepoint:* T1: six months postpartum T2: 12 months postpartum T3: 18 months postpartum	Pregnant women Thula Sana (n = 220) vs. no treatment (n = 229)	Living in a peri-urban area of Africa, in poor and overcrowded conditions No	SSP^(T3)^ CIS^(T2)^: maternal sensitivity and maternal intrusiveness	Six months postpartum sensitivity, *p* = 0.037*, *d* = 0.24; decreased intrusiveness, *p* = 0.024*, *d* = 0.26 12 months postpartum sensitivity,* p* = 0.043*, *d* = 0.26; decreased intrusiveness, *p* = 0.023*, *d* = − 0.24 18 months postpartum secure attachment, *p* = 0.029*
3	Cooper et al. (2002) South Africa Moderate 50%	Within-group *Single timepoint:* Six months postpartum	Pregnant women Thula Sana (n = 32) compared to data of a matched group (i.e., age/marital status) of mothers from an epidemiological sample in an adjacent area at the same time the intervention was being delivered (n = 32)	Living in a peri-urban area of Africa, in poor and overcrowded conditions No	Video-recorded free play and mother feeding baby coded using an eight-point Likert scale for overall sensitivity, the quality of the interaction and infants overall engagement in the interaction in play, interactive engagement and affective expression	Six months postpartum sensitivity, *p* = 0.02*; positive expression in the feeding task, *p* = 0.08*
	***UCLA Family Development Project***					
1	Heinicke et al. (2001) USA Good 66.6%	Independent groups comparison *Multi-timepoint:* T1: prenatally T2: infant at one month T3: infant at six months T4: infant at 12 months T5: infant at 24 months	Mother-infant dyads UCLA (n = 31) vs. treatment as usual (n = 33)	Identified as ‘at risk’ who were recruited in the original 1999 RCT study No	Home and laboratory observations coded using GRS for five caregiver dimensions (responsiveness to infant’s needs, encourages autonomy, encouragement in task involvement, intrusive play, positive affect) and five infant dimensions (expects care, sense of separate self, noncompliant play, positive affect)^(T2, T3, T4)^ BSID^(T3, T4, T5)^: response to separation/reunion	24 months caregiver dimensions responsiveness to infant’s needs, *p* = .0001*, *d* = 1.63; encourages autonomy, *p* = .01*, *d* = .67; encourages task involvement, *p* = .002*, d = 0.85; decreased intrusiveness, *p* = 0.01*,* d* = − 0.63; positive affect, *p* = 0.012*, *d* = 0.58; affectionate response to reunion, *p* = 0.005*, *d* = 1.0 24 months infant dimensions expectation to be cared for, *p* = 0.0001*,* d* = 2.0; sense of separate self, *p* 0.005*, *d* = 0.68; noncompliant play, *p* ≤ 0.001*,* d* = − 1.63; positive affect, *p* = 0.006*, *d* = 0.74; secure response to separation, *p* = 0.0001*, *d* = 1.31
2	Heinicke et al. (2000) USA Good 69.1%	Within-group *Multi-timepoint:* Each measure was administered once between the last trimester of pregnancy through to the infant being 12 months	Mothers-infant dyads and intervenors (n = 45; 31 RCT families from above study and 15 families independent of trial)	Identified as ‘at risk’ No	Home observations coded using GRS for mother’s responsiveness and efficiency in meeting the infant’s needs and the infant’s expectation of being cared for BSID: response to separation/reunion	Quality of partner support correlated with infant’s secure response to separation, *p* = 0.011* and expectation of care, *p* = 0.027* Mother’s ability to work with the intervenor correlated with responsiveness of their infant’s needs, *p* = 0.042* Mother’s ability to trust the intervenor correlated with the infant’s expectation of being cared for, *p* = 0.016*
3	Heinicke et al. (1999) USA Good 66.6%	RCT *Multi-timepoint:* T1: prenatally T2: infant at one month T3: infant at six months T4: infant at 12 months T5: infant at 14 months	Mother-infant dyads UCLA (n = 31) vs. treatment as usual (n = 33)	Identified as ‘at risk’ No	Home observations coded using GRS for five caregiver dimensions (responsiveness to infant’s needs, encourages autonomy, encouragement in task involvement, intrusive play, positive affect) and five infant dimensions (expects care, sense of separate self, noncompliant play, positive affect)^(T2, T3, T4)^ SSP^(T5)^ AQS^(T4)^ BSID^(T3, T4)^: response to separation/reunion	12 months caregiver dimensions responsiveness to infant’s needs, *p* = 0.0001*, *d* = 1.49; encourages autonomy, *p* = 0.0001*, *d* = 1.38; encourages task involvement, *p* = 0.019*, *d* = 0.55; decreased intrusiveness, *p* = 0.0012*,* d* = − 1.01; positive affect, *p* = 0.0017*, *d* = 0.82 12 months infant dimensions expectation of being cared for, *p* = 0.0001*, *d* = 1.37; sense of separate self, *p* = 0.0002*, *d* = 0.99; noncompliant play, *p* = 0.0046*, *d* = − 0.87, positive affect, *p* = 0.003*, *d* = 0.27 Both caregiver and infant found to have a more secure response to separation, *p* = 0.035*, *d* = 1.84 14 months secure attachment, *p* = 0.0209*; this was linked to caregiver positive affect
	***Watch,Wait and Wonder (WWW)***					
1	Cohen et al. (2002) Canada Good 64.2%	Independent groups comparison *Multi-timepoint:* T1: pre-intervention T2: post-intervention T3: six months follow up	Mother-infant dyads WWW (n = 26) vs. mother-infant psychotherapy group like WWW (n = 31)	Recruited from the 1999 study Yes	SSP CPS: reciprocity, intrusiveness, unresponsiveness and conflict	Six months follow-up reciprocity *p* = 0.05*,* d* = − 0.16; decreased intrusiveness *p* = 0.05*, *d* = − 0.13; only slight differences in unresponsiveness and conflict Six months follow-up secure attachment, 32% shifted/retained secure attachment classification vs. 36% in the control group
2	Cohen et al. (1999) Canada Good 69.1%	Independent groups comparison *Multi-timepoint:* T1: pre-intervention T2: post-intervention	Mother-infant dyads WWW (n = 34) vs. mother-infant psychotherapy group like WWW (n = 33)	Part of a regional mental health network for children ranging from infancy-adolescence Yes	SSP CPS: reciprocity, intrusiveness, unresponsiveness and conflict	Post-intervention reciprocity, *p* = 0.01*, *d* = − 0.36; decreased intrusiveness, *p* = 0.01*, *d* = − 0.03; decreased conflict, *p* = 0.01*, *d* = − 0.16 No significant group difference to unresponsiveness Intervention group more likely to move towards a secure or organised attachment post-intervention, *p* = 0.03*; 20.6% shifted to a secure attachment vs. 3% in the control group; 14.7% shifted from disorganised to insecure attachment vs. 9.3% in the control group

*Statistically significant finding; AAA, Adult Attachment Assessment (Hazan & Shaver, 1987); AAI, Adult Attachment Interview (Main et al., 1993, unpublished); AMBIANCE, Atypical Maternal Behaviour Instrument for Assessment and Classification (Lyons-Ruth et al., 1999); ARR, Assessment of Representational Risk (Sleed, 2013); AQS, Attachment Q-Sort (Waters, 1986); BSID, the Bayley Scales of Infant Development (Bayley, 1969); CEFR, Children’s Expression of Feeling in Relationships (Quinton et al., 1998); CIB, Coding Interactive Behaviour Scales (Feldman, 1998); CIS, Caregiver Involvement Scale (Farran et al*.*, cited in Cooper et al., 2009); CPS, The Chatoor Play Scale (Chatoor et al., 2018*)*; CRFQ, Caregiver Reflective Functioning Questionnaire (Ramsauer et al., 2014); EAS, Emotional Availability Scales (Biringen et al., 2010); ECR, Early Experiences in Close Relationships Scale (Brennan et al., 1998); GRS, Global Rating Scales; HOME, Home Observation for Measurement of the Environment (Bradley et al., 1977); IBS, Interactive Behaviour Scales (Ainsworth et al., 1978); KIPS, Keys to Interactive Parenting Scale (Comfort & Gordon, 2006); MAS, Maternal Attitude Scale (Cohler et al., 1970); MBQS, Maternal Behaviour Q-Sort (Pederson et al., 1990); MCS, Massie Campbell Scales (Massie & Campbell, 1992); MFAS, Maternal–Fetal Attachment Scale (Cranley, 1981); MORS, The Mothers Object Relations Scale (Milford & Oates, 2009); MPAAS, Maternal/Paternal Antenatal Attachment Scale (Condon, 1993); NCAST, Nursing Child Assessment Satellite Training (Farel et al., 1991); NICHD OCRE, National Institute of Child and Human Development [NICHD] Observational Record of Caregiving Environment (NICHD, 1996); PDI, Parent Development Interview (Slade et al., 2004a); PI, Pregnancy Interview (Slade et al., 2004a, 2004b); PRAQ, Pregnancy-Related Anxiety Questionnaire (Rohner, 2001); PRF, Parental Reflective Functioning Scale (Slade et al., 2004b); QRCI, Qualitative Rating for Parent–Child Interaction at 3-48 months (Mills-Koone & Cox, 2013, unpublished); SSP, Strange Situation Procedure (Ainsworth et al., 1978)

^a^Full RCT, either stated in the study title or method section, or if registration as a clinical trial is present; pilot RCT, if stated within the study title or method section

^b^Timepoint identified if different to that of design

^c^Effect sizes for statistically significant findings reported only,* d* = small (0.2–0.49), medium (0.5–0.79) and large (> 0.8)

### Overview of Relational Measures Used in Studies

The caregiver-infant relational outcomes measured were varied and included caregiver-infant attachment security, caregiver sensitivity/behaviours/interaction, caregiver reflective function, caregiver attachment representations and caregiver perceptions of the relationship. To evaluate these outcomes, observer-rated measures were predominantly used (n = 36, 90%) and 14 studies (35%) combined the observation with caregiver self-report measures (*ABC*: Berlin et al., 2018; *COS*: Røhder et al., 2022; Ramsauer et al., 2019; Cassidy et al., 2010, 2011; *GABI*: Myers et al., 2022; Steele et al., 2010; *Lighthouse MTB Parenting Programme*: Byrne et al., 2019; *MB*; Alhusen et al., 2020; *MTB*: Slade et al., 2020; Sadler et al., 2013; *New Beginnings*: Bain, 2014; Sleed et al., 2013; Mellow Babies: Raouna et al., 2021). Three studies (7.5%) adopted caregiver self-report measures related to the caregiver-infant relationship only (*COS*: Maxwell et al., 2020; *The Solihull Approach*: Douglas & Bateson, 2017; Harris-Waller et al., 2019).

Of the 19 studies (47.5%) that evaluated caregiver-infant attachment, 14 studies utilised The Strange Situation Procedure (Ainsworth et al., 1978) and two studies used the Attachment Q-Sort (Waters, 1986; *RFTS*: Niccols, 2008; *UCLA Family Development Project*: Heinicke et al., 1999). Other measures used in studies included the Massie-Campbell Scales (Massie & Campbell, 1992; *Strengthening Relationships Towards Secure Attachment:* Leigh et al., 2013), the Maternal/Paternal Antenatal Attachment Scale (Condon, 1993; *The Solihull Approach*: Douglas & Bateson, 2017) and the Maternal–Fetal Attachment Scale (Cranley, 1981; *MB*: Alhusen et al., 2020).

Twenty-six studies (65%) evaluated various qualities of the caregiving interaction and/or behaviours (i.e., sensitivity, responsiveness, intrusiveness, delight, control, regard, warmth, hostility etc.) using numerous standardised measures. Three studies (7.5%) developed Likert rating scales specifically for the purpose of the study. Caregiver sensitivity was evaluated standalone in five studies using either study-specific Likert rating scale (*ABC*: Bick & Dozier, 2013; *COS*: Cassidy et al., 2010) or standardised measure (Maternal Behaviour Q-Sort, Pederson et al., 1990; *ABC*: Berlin et al., 2014; *COS*: Ramsaeur et al., 2019; *RFTS*: Niccols, 2008). Caregiver reflective functioning was evaluated in seven studies (17.5%). Six studies used the Parent Development Interview (Slade et al., 2004a; *Lighthouse MBT Parenting Programme*: Byrne et al., 2019; *MTB*: Slade et al., 2020; Sadler et al., 2013; *New Beginnings*: Bain, 2014; Sleed et al., 2013).

Caregiver attachment representations and styles were evaluated in six studies (15%) using standardised measures including the Early Experiences of Close Relationships Scale (ECR; Brennan et al., 1998) in two studies (*COS*: Cassidy et al., 2010, 2011) and the Adult Attachment Interview (Main et al., 1998, unpublished manuscript)) in three studies (*COS*: Ramsauer et al., 2019; *GABI*: Steele et al., 2010; Myers et al., 2022). One study used the Maternal Attitude Scale (Cohler et al., 1970). Berlin et al., (2018, *ABC*) used two evaluative measures of caregiver attachment including the ECR (Brennan et al., 1998) and the Adult Attachment Assessment (Hazan & Shaver, 1987). Caregiver perceptions of the relationship with the infant were evaluated in one study (*New Beginnings*: Sleed et al., 2013), using the standardised Mothers Object Relations Scale (Milford & Oates, 2009). The Children’s Expression of Feeling in Relationships (Quinton et al., 1998) was used to evaluate the caregivers' perceptions of the relationship with the infant in one study (*The Solihull Approach*: Harris-Waller et al., 2019).

### Reported Findings of Differences to Caregiver-Infant Relational Processes

Across the 16 included interventions, 32 studies (80%) reported a statistically significant positive change in an investigated caregiver-infant relational outcome for the intervention compared with either pre-intervention or a control group(s). One study included no pre-intervention measures or comparison group to enable evaluation of difference (*GABI*: Steele et al., 2010). Four studies reported improvements compared to controls through descriptive statistics only (*ABC*: Caron et al., 2016; *COS*: Cassidy et al., 2011; *Secure Attachment Promotion Program*, Santelices et al., 2011; *Strengthening Relationships Towards Secure Attachment*, Leigh et al., 2013). Several studies reported suggestive relational findings which did not reach statistical significance: Berlin et al., (2014, *ABC*); Ramsaeur et al. (2019) and Røhder et al., (2022, *COS*), and Harris-Waller et al., (2019, *The Solihull Approach*). Statistically significant findings are described below.

Nine studies reported a significantly higher rate of secure caregiver-infant attachment at post-intervention when compared to control(s) (*ABC*: Bernard et al., 2012; *COS*: Cassidy et al., 2010; *MB*: Alhusen et al., 2020; *MTB*: Slade et al., 2020; Sadler et al., 2013; *SAFE*: Walter et al., 2019; *Thula Sana*: Cooper et al., 2009; *UCLA Family Development Project*: Heinicke et al., 1999; *WWW*: Cohen et al., 1999). One study reported a significant pre- to post-intervention difference in secure attachment (*The Solihull Approach*: Douglas & Bateson, 2017). Niccols (2008, *RFTS*) reported that infant attachment change scores were correlated with caregiver sensitivity over time for the intervention group only.

Twenty-six (65%) studies reported caregivers to demonstrate significantly more positive interactions/behaviours towards infants following the intervention compared to control(s). Seven studies reported differences in one domain (*ABC*: Perrone et al., 2020; Yarger et al., 2019; Berlin et al., 2014; Bick & Dozier, 2013; *Lighthouse MBT Parenting Programme*: Byrne et al., 2019; *MTB*: Slade et al., 2020; *New Beginnings*: Bain, 2014) and 17 reported multiple significant differences to caregiving behaviours (*ABC*: Harden et al., 2021; Berlin et al., 2018; Yarger et al., 2016; *COS*: Maxwell et al., 2020; Myers et al., 2022; *GABI*: Steele et al., 2019; *Mellow Babies*: Puckering et al., 2010; Raouna et al., 2021; *MB*: McFarlane et al., 2017; *MTB*: Sadler et al., 2013; *New Beginnings*: Sleed et al., 2013; *Thula Sana*: Valades et al., 2021; Cooper et al., 2002, 2009; *UCLA Family Development Project*: Heinicke et al., 1999, ; *WWW*: Cohen et al., 1999, 2002).

Caregiver reflective function was significantly improved in three studies compared to controls (*MTB*: Slade et al., 2020; Sadler et al., 2013; *New Beginnings*: Sleed et al., 2013). Sadler et al., (2013, *MTB*) emphasised changes specifically to caregiver mentalising. Three studies reported on the influence of caregiver attachment status in determining caregiver-infant relational outcomes (*ABC*: Berlin et al., 2018; *COS*: Ramsauer et al., 2019; Cassidy et al., 2010). One study reported significant positive changes to caregiver perception of the relationship with the infant from pre- to post-intervention (*New Beginnings*: Sleed et al., 2013).

All reported effect sizes for statistically significant relational findings were calculated or recalculated using the Morris (2008) method and reported according to Cohen’s *d* (Cohen, 1992; difference between means/pooled standard deviations). They reflected the standardised difference in mean scores of relational measure constructs between the intervention group and the control group over time, or the intervention group from pre- to post-intervention. In line with Cohen (1992), effect sizes were defined as small (0.2–0.49), medium (0.5–0.79) and large (> 0.8). Overall, effect sizes for intervention on the caregiver-infant relationship were varied within and across studies, ranging from large positive to large negative (e.g., *ABC*: Yarger et al., 2016; *Mellow Babies*: Puckering et al., 2010).

### Additional Measures and Findings

A table summarising additional measures and findings is presented in Appendix G. Thirty-four of the included studies (85%) explored additional outcomes to caregiver-infant relational processes, which included infant development and behaviour (n = 9), caregiver psychopathology (n = 24; i.e., depression, anxiety, trauma, dissociation), caregiver functioning (n = 14; i.e., overall competence and mastery in activities of daily living, stress), caregiver social support (n = 3), caregiver and infant physical and cognitive health (n = 2) and experiences of the program/alliance with the therapist (n = 8). Standardised, well-validated measures were predominantly incorporated (n = 44) and 13 studies utilised a measure that was designed specifically for the study. Sixteen studies reported a statistically significant finding of outcomes additional to the relational dyad examined.

A significant reduction in depression symptoms was most frequently reported following intervention (n = 8; *COS*: Maxwell et al., 2020; Cassidy et al., 2010; Røhder et al., 2022; *Mellow Babies*: Puckering et al., 2010; *MB*: McFarlane et al., 2017; *MTB*: Slade et al., 2020; *WWW*: Cohen et al., 1999). *UCLA Family Development Project* evaluated caregiver social support in two included studies and was reported to improve significantly following the intervention at 12 months (Heinicke et al., 1999) and 24 months (Heinicke et al., 2001). Interventions were found to have significant positive effects on infant physical and cognitive health, performance or development in eight studies (*ABC*: Harden et al., 2021; *MTB*: Sadler et al., 2013; *New Beginnings*: Bain, 2014; *The Solihull Approach*: Harris-Waller et al., 2019; *Thula Sana*: Cooper et al., 2002; *WWW*: Cohen et al., 1999, 2002).

## Discussion

This systematic literature review was the first comprehensive synthesis of manualised attachment-based interventions for caregivers and infants from conception to two years focussing on relational outcomes. Initially, 26 eligible manualised attachment-based interventions were identified, then after searching empirical studies evaluating caregiver-infant relational components, 40 studies supporting 16 interventions were identified. By contrast, no eligible evidence was located for ten interventions mainly because either the participating children were over two years old, the sample proportion of children over two years was not confirmed by the author, or the outcomes did not include a relational measure of the dyad. It is important to note that the non-inclusion of these studies is not necessarily a reflection on the intervention themselves, but rather on the fact that there was limited information or evidence relevant for this review.

A key overall finding of this review was that 16 manualised attachment-based interventions were each found to have some evidence that supported their effectiveness in improving at least one caregiver-infant relational process (i.e., caregiver-infant attachment, caregiver interactions/behaviours, caregiver reflective function, caregiver attachment representations, caregiver perceptions of the relationship with the infant). Whilst previous reviews have determined the effectiveness of attachment-based interventions based on the type of intervention implemented (Bakermans-Kranenberg et al., 2003, 2005; Broberg, 2000; Cornell & Hamrin, 2008; Facompré et al., 2018; Kerr & Cossar, 2014), the current review offers novel findings by considering intervention effectiveness based on the combined evidence available for an eligible intervention. Furthermore, findings also revealed the variability of empirical evidence supporting these 16 interventions leading to improved relational outcomes, in terms of number, quality and design. Considering previous review findings, the effectiveness of the included interventions on improving caregiver-infant relational functioning was not surprising, but the success of interventions reviewed here are subject to deliberation. One explanation may be the influence of social variables on families that may benefit relational outcomes. It is worth considering how some interventions included in this review specifically target families who were ‘at risk’ or had experienced adversity (e.g., *ABC*, *COS*, *GABI*, *New Beginnings*). Although there is some evidence to suggest that such families may have difficulty in engaging with an intervention due to the adversity being faced (Bentovim et al., 2019), it is worth considering how families may be more susceptible to respond positively to support that has previously been absent and is very much needed and consequently demonstrate significant change.

The review findings highlighted the predominant use of observational methods of the caregiver and infant, with few studies using only caregiver self-report measures (*COS*: Maxwell et al., 2020; *The Solihull Approach*: Douglas & Bateson, 2017; Harris-Waller et al., 2019). This finding was unexpected and offers contradictory evidence to Broberg’s (2000) suggestion that attachment-based intervention outcomes are often reported through parental self-report measures. Behavioural observation, as the “natural starting point” for assessment and intervention between a caregiver and infant (O’Connor & Zenah, 2003, p. 229), has played a prominent role in caregiver-infant research (Hawes & Dadds, 2006), because observations allow researchers to precisely understand, recognise and record inferences between interactions, relationships and behaviour (Pellegrini, 2004). Thus, this review offers robust evidence that is based on rich, accurate data.

*ABC* was found to have the most studies investigating caregiver-infant relational outcomes. The most frequently reported positive outcome across *ABC* studies was caregiver sensitivity, which was determined to be significantly improved in four studies (Berlin et al., 2018; Bick & Dozier, 2013; Perrone et al., 2020; Yarger et al., 2016). Bick and Dozier (2013) reported improved caregiver sensitivity in their long-term follow-up at 24 months. These findings are encouraging for *ABC* because a core hypothesis of attachment theory is the crucial role of caregiver sensitivity in shaping positive infant outcomes (Gillath et al., 2016). *ABC* was also identified to be the intervention with the most RCT evidence (n = 7). RCT studies are classified as the highest level of evidence (Canadian Task Force, 1979) because this design minimises risk of bias and systematic error (Burns et al., 2011; Guyatt et al., 2008). This finding offers potential to the *ABC* intervention once again. However, *ABC* studies were all conducted in the USA, the rated study quality was variable (high = 1, good = 7, moderate = 1; Table 3) and the included studies predominantly compared *ABC* against the same intervention (Developmental Education for Families or ‘Book of the week’, n = 7). For instance, ABC was reported as being implemented by only 1.5% (*n* = *11*) of 625 services that work with children (aged 0–13 years) in the UK (Wright et al., 2023). Ideally, to ensure true effectiveness of an intervention, multiple studies using multiple and different comparison groups across different contexts, cultures and settings should be conducted (Parkhurst & Abeysinghe, 2016).

As shown in Table 3, many of the studies in this review were rated as good (n = 27) 67.5%) or high-quality (n = 10, 25%), which was contradictory to existing reviews that have reported attachment-based intervention studies are generally of poor quality (Drozd et al., 2018; Kerr & Cossar, 2014; Wright et al., 2017). However, Fenton et al. (2015) assumed that the QATSDD may produce inflated quality scores due to the criteria items included (and excluded) in the tool which may explain this finding. Furthermore, the difference in quality rating scores between the current review and previous reviews may be explained because existing reviews included studies that were only RCT and/or group comparison designs (Drozd et al., 2018; Wright et al., 2017), or included separate quality assessment checklists that were specific to study design (i.e., Kerr & Cossar, 2014, used an RCT checklist and cohort checklist).

Based upon the quality criteria outlined here, *MTB* appears promising because the intervention consisted of only high-quality RCT evidence. It is worth noting that only two studies were eligible for inclusion for *MTB*, but both studies reported positive intervention relational outcomes. The intervention, which was found to have the most high quality rated studies, was *COS* (n = 4). When considering all the included studies for *COS*, the intervention was found to report positive outcomes with regards to relational functioning as well as being one of the most cross-culturally implemented (Australia, Germany, Denmark and USA). Taken together, these findings may suggest that *COS* is based upon robust research evidence. However, some caution is required across *COS* study findings as samples were unequal (Maxwell et al., 2020) or small (Cassidy et al., 2010; Ramsauer et al., 2019), which may give the illusion of favourable intervention outcomes (Rusticus & Lovato, 2014; Sullivan & Feinn, 2012).

### Strengths and Limitations of this Review

The strengths of this systematic review lie in its rigorous, structured and replicable processes, through which multiple interventions and studies were explored and the findings synthesised. The review validity was increased by using an independent second reviewer during screening, quality appraisal and data extraction phases.

Given that both search strategies for Stages 1 and 2 were limited to the English language and sources published in peer-reviewed journal articles, language, location and selection biases need to be acknowledged. Whilst most of interventions and studies included were from the UK or USA, a variety of other countries and cultures were identified and included. In addition, this review highlights that various outcome measures have been used in the included studies, which is not uncommon in reviews of this kind. However, this diversity means that it is difficult to explore the effectiveness of any intervention by conducting a meta-analysis. Furthermore, it has been debated that only studies of the same design can be effectively synthesised (Arditi et al., 2016; Concato et al., 2010; Vandenbroucke, 2008), thereby a potential limitation of this review is the inclusion of multiple study designs. However, the inclusion of more than one study design was deemed necessary to effectively answer the review questions and offer a comprehensive overview of the evidence-base. Furthermore, guidance on conducting systematic reviews of interventions encourages the inclusion of diverse study designs to allow for an in-depth understanding and context of the intervention (Craig et al., 2008).

### Clinical Implications

A key clinical implication of this review is that it provides a clear overview of available manualised attachment-based interventions that do not have a core component of video-feedback. The overview includes practical and contextual information on intervention aims, format, delivery, target groups, manualisation and training. Consequently, it allows healthcare professionals, commissioners and policymakers within perinatal sectors the consideration of training and implementation of a range of manualised attachment-based interventions.

Beyond this, the review also offers an outline of each of these interventions evidence-base in relation to improving the caregiver-infant dyadic relationship. There was promising evidence determined from the current review for *ABC*, *COS* and *MTB* which services may wish to consider implementing. However, it is important to note that these interventions were not without their empirical methodological limitations. Although not limited to the perinatal period, Wright et al’s (2023) survey and systematic review of routinely used interventions to improve disorganised attachment in children (aged 0–13 years) reported a disparity between evidence-based research and clinical implementation. Services are implementing interventions that at present have limited evidence to support their use. Therefore, as Wright et al. (2023) suggests, collaborations between researchers and practitioners are warranted to ensure the most effective interventions for the caregiver-infant dyad are being implemented in services.

### Future Research

Firstly, it is suggested that more funding may need to be invested in enabling interventions to conduct more RCTs to build on the accuracy of the findings reported in this review and enhance the effectiveness of included interventions (Hariton & Locascio, 2018). Additionally, studies that adopt designs using diverse cross-cultural, socio-demographic contexts and samples would contribute towards building a stronger evidence-base for an intervention and allow for better generalisability of reported findings (Parkhurst & Abeysinghe, 2016).

To establish intervention efficacy further, future studies may need to invest in improving their methodological quality (Parkhurst & Abeysinghe, 2016). Based upon the current review’s quality appraisal findings, future researchers should include a power calculation for sample sizes because this is important to draw accurate and precise conclusions about the intervention (Nayak, 2010). Future studies may also benefit from the involvement of service users in their design because doing so may not only improve study quality but may also improve intervention quality by making it more relevant to those accessing it (Nielsen, 2013).

Furthermore, it is noteworthy that most of the samples in this review included mother-infant dyads and very few studies considered samples with fathers (with the exception of *SAFE*: Walter et al., 2019; *The Solihull Approach*: Douglas & Bateson, 2017), or with co-mothers or co-fathers. Given the positive associations between infant outcomes and relationships within the first two years, there is an urgency for interventions that successfully promote parent-infant bonding across different family formations (Bronte-Tinkew et al., 2008; Garfield & Issaco, 2006; Scism & Cobb, 2017). Thus, in future studies, it would be important to establish the effectiveness of the included interventions on multiple and diverse dyads as well as mother-infant dyads.

Finally, it may be relevant to focus on implementation aspects in a future review of such interventions, with attention being paid to fidelity assessments and appropriate supervision arrangements to avoid intervention drift in practitioners.

### Conclusions

This systematic review adds to the growing literature exploring the effectiveness of attachment-based interventions. It contributes novel findings by focussing on the level of evidence of manualised attachment-based interventions related to aspects of caregiver-infant relational functioning. Findings suggest that currently available and accessible manualised attachment-based interventions vary greatly in the amount and quality of empirical evidence supporting caregiver-infant relational change. Clinicians and services who implement attachment-based interventions into practice should be guided towards the interventions that have been suggested to have the most encouraging supporting evidence. However, more high-quality studies are needed before firm conclusions can be made.

## Supplementary Information

Below is the link to the electronic supplementary material.

Supplementary file1 (DOCX 287 KB)

## Footnotes

1 Intervention names have only been abbreviated in this review if and how they have been abbreviated in the wider literature.
