# Supplementary material for: Manualised Attachment-Based Interventions for Improving Caregiver-Infant Relationships: A Two-Stage Systematic Review
Source: Clin Child Fam Psychol Rev. 2024 Nov 18;28(1):71–100. doi: 10.1007/s10567-024-00497-0 (PMC11885378; doi:10.1007/s10567-024-00497-0)
Supplement: Supplementary file 1 — Supplementary file1 (DOCX 287 KB) [file 10567_2024_497_MOESM1_ESM.docx]

**APPENDIX A: Stage 1 search strategy**

| **Database search** | **Search term** | **Items found** |
| --- | --- | --- |
| **CINAHL Plus (EBSCO)** | | |
| #1 | “attachment*” OR “attachment-based” | 20,269 |
| #2 | “intervention” OR “group” OR “programme” OR “program” or “treatment” | 2,318,609 |
| #3 | (S1 AND S2) | 9,153 |
| #4 | “parent*” OR “mother” OR “father” OR “caregiver” | 193,693 |
| #5 | “child” OR “toddler” OR “infant” OR “preschool*” OR “baby” | 906,938 |
| #6 | (S4 AND S5) | 145,172 |
| #7 | (S3 AND S6) | 1,716 |
| **EMBASE 1974 to 2020 January 08 (OVID)** | | |
| #1 | “attachment*” OR “attachment-based” | 128,271 |
| #2 | “intervention” OR “group” OR “programme” OR “program” or “treatment” | 10,515,521 |
| #3 | (S1 AND S2) | 37,339 |
| #4 | “parent*” OR “mother” OR “father” OR “caregiver” | 586,421 |
| #5 | “child” OR “toddler” OR “infant” OR “preschool*” | 2,523,853 |
| #6 | (S4 AND S5) | 268,584 |
| #7 | (S3 AND S6) | 2,234 |
| **Medline 1946 to December week 5 2019 (OVID)** | | |
| #1 | “attachment*” OR “attachment-based” | 107,743 |
| #2 | “intervention” OR “group” OR “programme” OR “program” or “treatment” | 6,521,673 |
| #3 | (S1 AND S2) | 28,155 |
| #4 | “parent*” OR “mother” OR “father” OR “caregiver” | 421,040 |
| #5 | “child” OR “toddler” OR “infant” OR “preschool*” | 2,566,130 |
| #6 | (S4 AND S5) | 198,701 |
| #7 | (S3 AND S6) | 1,618 |
| **PsycINFO 1806 to January week 1 2020 (OVID)** | | |
| #1 | “attachment*” OR “attachment-based” | 45,503 |
| #2 | “intervention” OR “group” OR “programme” OR “program” or “treatment” | 1,428,798 |
| #3 | (S1 AND S2) | 13,700 |
| #4 | “parent*” OR “mother” OR “father” OR “caregiver” | 297,164 |
| #5 | “child” OR “toddler” OR “infant” OR “preschool*” | 507,623 |
| #6 | (S4 AND S5) | 168,736 |
| #7 | (S3 AND S6) | 3,331 |
| **Web of Science (1900-2020)** | | |
| #1 | “attachment*” OR “attachment-based” | 183,642 |
| #2 | “intervention” OR “group” OR “programme” OR “program” or “treatment” | 10,745,103 |
| #3 | (S1 AND S2) | 49,774 |
| #4 | “parent*” OR “mother” OR “father” OR “caregiver” | 578,300 |
| #5 | “child” OR “toddler” OR “infant” OR “preschool*” | 1,919,177 |
| #6 | (S4 AND S5) | 227,381 |
| #7 | (S3 AND S6) | 3,619 |
| **Total** | | |
| Total records |  | 12,518 |

**APPENDIX B: Brief overview of attachment-based interventions identified as using video-feedback as a core component**

| **Video-based interventions with a clear name** | |
| --- | --- |
| **1** | **Attachment Video-Feedback Intervention (AVI)**  Tarabulsy, G. M., Baudry, C., Pearson, J., & Turgeon, K. (2016). *An evaluation of the implementation of an attachment-based intervention strategy within child-protection services.* National Institute for Excellence in Health and Social Services, Ministry of Health and Social Services. |
| **2** | **Make the Connection (MTC)**  The Psychology Foundation (year unknown). No reference available |
| **3** | **Promoting First Relationships**  Kelly, JF., Zuckerman, TG., & Rosenblatt, S. (2008). Promoting First Relationships: A relationship-focused early intervention approach. *Infants and Young Children, 4*(21), 285–295. <https://doi.org/10.1097/01.IYC.0000336541.37379.0e>. |
| **4** | **Promoting Responsiveness, Emotion Regulation and Attachment in Young Mothers and Infants (PRERAYMI)**  Riva Crugnola, C., Ierardi, E., Peruta, V., Moioli, M., & Albizzati, A. (2021). Video-feedback attachment-based intervention aimed at adolescent and young mothers: Effectiveness on infant-mother interaction and maternal mind-mindedness. *Early Child Development and Care, 191*(3), 475-489. <https://doi.org/10.1080/03004430.2019.1652172>. |
| **5** | **Steps Towards Effective Enjoyable Parenting (STEEP)**  Erickson, M.F., & Egeland, B. (2004). Linking theory and research to practice: The Minnesota longitudinal study of parents and children and the STEEP™ program. Clinical Psychologist, *8*(1), 5–9. <https://doi.org/10.1080/13284200410001672207>. |
| **6** | **ULM Model (“A Good Start to Life”)**  Pillhofer, M., Spangler, G., Bovenschen, I., Kuenster, A. K., Gabler, S., Fallon, B., Fegert, J.M and Ziegenhain, U. (2015). Pilot study of a program delivered within the regular service system in Germany: Effect of a short-term attachment-based intervention on maternal sensitivity in mothers at risk for child abuse and neglect. *Child Abuse & Neglect*, *42*, 163-173. <https://doi.org/10.1016/j.chiabu.2014.07.007>. |
| **7** | **Video Interactive Guidance (VIG)**  Biemans., H. (1990). Video home training: theory, method and organization of SPIN. In J. Kool (Eds). *International seminar for innovative institutions*. Ministry of Welfare Health and Culture. |
| **8** | **Video-Feedback Intervention to Promote Positive Parenting (VIPP)**  Juffer, F., Bakermans‐Kranenburg, M. J., & van Ijzendoorn, M. H. (2005). The importance of parenting in the development of disorganized attachment: Evidence from a preventive intervention study in adoptive families. *Journal of Child Psychology and Psychiatry*, *46*(3), 263-274. <https://doi.org/10.1111/j.1469-7610.2004.00353.x>.  Juffer, F., Bakermans-Kranenburg., M.J., & van Ijzendoorn., M.H. (2008). *Promoting* positive parenting: An attachment-based intervention. Routledge*.*  Juffer, F., Hoksbergen, R.A.C., Riksen‐Walraven, J.M. and Kohnstamm, G.A. (1997). Early intervention in adoptive families: Supporting maternal sensitive responsiveness, infant-mother attachment, and infant competence. *Journal of Child Psychology and Psychiatry, 38*(8), 1039-1050. <https://doi.org/10.1111/j.1469-7610.1997.tb01620.x>.  There were many variations of VIPP identified, including:   - Video-Feedback Intervention to Promote Positive Parenting-Sensitive Discipline (VIPP-SD) - Video-Feedback Intervention to Promote Positive Parenting-Representational Focus (VIPP-R) - Video-Feedback Intervention to Promote Positive Parenting in Foster Care (VIPP-FC) - Video-Feedback Intervention to Promote Positive Parenting for Family Placements (VIPP-FP) - Video-Feedback Intervention to Promote Positive Parenting-Learning Difficulties (VIPP-LD) - Video-Feedback Intervention to Promote Positive Parenting adapted to autism (VIPP-AUTI) - Video-Feedback Intervention to Promote Positive Parenting-Visual (VIPP-V) - Video-Feedback Intervention to Promote Positive Parenting-Child Care (VIPP-CC) - Video-Feedback Intervention to Promote Positive Parenting for Co-Parents (VIPP-Co) |
| **9** | **Video Intervention Therapy (VIT)**  Downing (2015). No reference available |
| **Video-based interventions without a clear name** | |
| **1** | Moran, G., Pederson, D. R., & Krupka, A. (2005). Maternal unresolved attachment status impedes the effectiveness of interventions with adolescent mothers. *Infant Mental Health Journal, 26*(3), 231-249. <https://doi.org/10.1002/imhj.20045>. |
| **2** | Moss, E., Dubois-Comtois, K., Cyr, C., Tarabulsy, G.M., St-Laurent, D., & Bernier, A. (2011). Efficacy of a home-visiting intervention aimed at improving maternal sensitivity, child attachment, and behavioral outcomes for maltreated children: A randomized control trial. *Development and Psychopathology, 23*(1), 195-210. <http://dx.doi.org/10.1017/S0954579410000738>. |
| **3** | Stams, G.‐J.J.M., Juffer, F., van Ijzendoorn, M.H., & Hoksbergen, R.C. (2001). Attachment‐based intervention in adoptive families in infancy and children's development at age 7: Two follow‐up studies. *British Journal of Developmental Psychology, 19*(2), 159-180. <https://doi.org/10.1348/026151001166010>. |
| **4** | Svanberg, P.O., Mennet, L., & Spieker, S. (2010). Promoting a secure attachment: A primary prevention practice model. *Clinical Child Psychology and Psychiatry*, *15*(3), 363-378. <http://dx.doi.org/10.1177/1359104510367584>. |
| **5** | van Doesum, K. T., Riksen‐Walraven, J. M., Hosman, C. M., & Hoefnagels, C. (2008). A randomized controlled trial of a home‐visiting intervention aimed at preventing relationship problems in depressed mothers and their infants. *Child Development*, *79*(3), 547-561. <http://dx.doi.org/10.1111/j.1467-8624.2008.01142.x>. |

**APPENDIX C: Stage 2 original search strategy from June 2020 (PsycINFO 1806 – June week 1 2020; OVID)**

| **Database search** | **Search term** | **Items found** |
| --- | --- | --- |
| #1 | “Attachment*” OR “Relationship” OR “Interaction” OR “Dyad” OR “Bond” OR “Sensitivity” OR “Responsiveness” OR “Attunement” OR “Reflexivity” | 891,312 |
| #2 | “Attachment and biobehavioural catch-up” OR “ABC” | 2729 |
| #3 | S1 AND S2 | 559 |
| #4 | “Baby Bonding” OR “Big Toes Little Toes” | 16 |
| #5 | S1 AND S4 | 12 |
| #6 | “Circle of Security” OR “COS-P” OR “Circle of Security Parenting” “OR “COS-I” | 64 |
| #7 | S1 AND S6 | 63 |
| #8 | “First Play Therapy” | 2 |
| #9 | S1 AND S8 | 0 |
| #10 | “Foundation for Attachments Group” | 32 |
| #11 | S1 AND S10 | 32 |
| #12 | “Group Attachment Based Intervention” OR “GABI” | 25 |
| #13 | S1 AND S12 | 15 |
| #14 | “Lighthouse MBT Parenting Programme” | 1 |
| #15 | S1 AND S14 | 1 |
| #16 | “Mellow Babies” | 6 |
| #17 | S1 AND S16 | 3 |
| #18 | “Mellow Bumps” | 2 |
| #19 | S1 AND S18 | 2 |
| #20 | “Mellow Parenting” | 17 |
| #21 | S1 AND S20 | 12 |
| #22 | “Minding the baby” OR “MTB” | 22 |
| #23 | S1 AND S22 | 18 |
| #24 | “Mom Power” | 6 |
| #25 | S1 AND S24 | 5 |
| #26 | “Mothers and Toddlers Program” OR “MTP” | 4 |
| #27 | S1 and S26 | 4 |
| #28 | “New Beginnings” | 219 |
| #29 | S1 AND S28 | 62 |
| #30 | “Nurturing Attachments Group” | 0 |
| #31 | S1 AND S30 | 0 |
| #32 | “Parent-Infant Psychotherapy” OR “Infant-Parent Psychotherapy” OR “PIP” | 171 |
| #33 | S1 AND S32 | 156 |
| #34 | “Right From the Start” OR “RFTS” | 185 |
| #35 | S1 AND S34 | 31 |
| #36 | “Secure Attachment Family Education Prevention Program” OR “SAFE Prevention Program” | 29 |
| #37 | S1 AND S36 | 3 |
| #38 | “Secure Attachment Promotion Programme” | 0 |
| #39 | S1 AND S38 | 0 |
| #40 | “Strengthening Relationships Towards Secure Attachment” | 0 |
| #41 | S1 AND S40 | 0 |
| #42 | “The Solihull Approach” OR “Understanding Your Child’s Behaviour” OR “UYCB” | 10 |
| #43 | S1 AND S42 | 10 |
| #44 | “Theraplay” | 140 |
| #45 | S1 AND S44 | 97 |
| #46 | “Thula Sana” | 1 |
| #47 | S1 AND S46 | 0 |
| #48 | “UCLA Family Development Project” | 10 |
| #49 | S1 AND S48 | 10 |
| #50 | “Watch, Wait and Wonder” OR “WWW” | 23 |
| #51 | S1 AND S50 | 20 |
| **Total** | | |
| Total records |  | 1,115 |

**APPENDIX D: Excluded interventions (Stage 1) with reasons**

| ***Interventions with a clear name*** | | |
| --- | --- | --- |
|  | **Intervention, brief description, developer(s), year and reference** | **Exclusion reason** |
| **1** | **BASE Babywatching**  A school-based, teacher-led programme where a parent and their baby, visit a class of children. The children watch the interactions between mother and baby and their developing relationship  **Developer:** Brisch (2007)  **Reference:** Brisch, K. H. (2007). *B.A.S.E. - Babywatching. A programme to prevent aggressive and anxious developmental disorders in toddlers*. Wiss. Symposium of Child Psychotherapy, Tauranga/Neuseeland, 26. Februar. | Intervention does not meet inclusion criteria for definition of an attachment-based intervention. This intervention is an educational programme. |
| **2** | **CAPEDP**  Aims to reduce infant disorganisation and maternal disorganising behaviour to promote secure attachment between caregiver and infant  **Developers:** Saïas et al (2013)  **Reference:**  Saïas, T., Greacen, T., Tubach, F., Dugravier, R., Marcault, E., Tereno, S., Tremblay, R.E., Guédeney, A. & CAPEDP Study Group. (2013). Supporting families in challenging contexts: the CAPEDP project. *Global Health Promotion*, *20*(2_suppl), 66-70. <https://doi.org/10.1177/1757975913483335>. | Intervention does not meet the inclusion criteria because the developer confirmed that there is no manual available. |
| **3** | **Child Parent Relationship Therapy (CPRT)**  A play therapy-based treatment program for young children presenting with behavioral, emotional, social, and attachment disorders  **Developers:** Landreth and Bratton (2006)  **Reference:**  Landreth, G. & Bratton, S. (2006). *Child parent relationship therapy (CPRT): A 10-session filialtherapy model*. Taylor & Francis. | Intervention does not meet inclusion criteria due to child target age range is above two years. |
| **4** | **Connect**  Aims to reduce child externalizing and internalizing problems, caregiver strain and depressed mood, increase parenting/family satisfaction and parent-child attachment security by promoting parenting sensitivity, reflective function, dyadic affect regulation and mutuality  **Developers:** Moretti et al (2004)  **Reference:**  Moretti, M. M., Holland, R., Moore, K., & McKay, S. (2004). An attachment-based parenting program for caregivers of severely conduct disordered adolescents: Preliminary findings. *Journal of Child and Youth Care Work, 19*, 170-79. | Intervention does not meet inclusion criteria due to child target age range is above two years. |
| **5** | **Dyadic Developmental Psychotherapy**  A therapist-led intervention that provides a framework for supporting looked after and adopted children to recover from trauma through the parenting and support. Requires the clinician to become affectively attuned with the child and caregivers, and to develop and maintain a meaningful emotional connection with the child  **Developer:** Hughes (year unknown)  **Reference:** No reference available | Intervention does not meet inclusion criteria due to child target age range is above two years. |
| **6** | **Family Links Nurturing Programme**  Aims to develop a positive relationship through appropriate expectations and boundaries, develop self-awareness, enhance self-esteem and teach children values  **Developer:** The Family Nurturing Network (based on the work of Stephen Bavolek)  **Reference:** No reference available | Intervention does not meet inclusion criteria for definition of attachment intervention. The intervention focuses on parenting strategies and has a strong social learning theory focus. |
| **7** | **Family Nurse Partnership (FNP – ‘Building Blocks’)**  Aims to improve adolescent pregnancy outcomes, improve child health and development and improve parents’ economic self-sufficiency  **Developer:** Olds (1986; 0riginated in USA as Nurse Family Partnership) and has moved to the UK and run by the Neonatal Unit UL  **Reference:**  Olds, D.L. (2006). The nurse–family partnership: An evidence‐based preventive intervention. *Infant Mental Health Journal*, *27*(1), 5-25. <https://doi.org/10.1002/imhj.20077>. | Intervention does not meet the inclusion criteria because developers did not confirm either the presence of a manual or if the intervention fundamentally targets the caregiver-infant relationship. |
| **8** | **Fostering Attachments Group**  Helps foster parents to develop their knowledge and understanding of the needs of children with attachment difficulties and reducing some of the stress they experience  **Developers:** Golding and Picken (2004)  **Reference:**  Golding, K., & Picken, W. (2004). Group Work for Foster Carers Caring for Children with Complex Problems. *Adoption & Fostering, 28*(1), 25-37. <https://doi.org/10.1080/14616739900134021>. | Intervention does not meet inclusion criteria due to child target age range is above two years. |
| **9** | **Fostering Changes Programme**  Aims to enhance carers’ relationships with their foster children by coaching them to respond sensitively to their child’s needs primarily through reflective listening and problem-solving  **Developer:** Adoption and Fostering National Team at Maudsley Hospital (year unknown)  **Reference:** No reference available | Intervention does not meet inclusion criteria due to child target age range is above two years. |
| **10** | **Kangaroo Care**  A method of caring for low-weight or premature babies outside an incubator. It involves strapping the baby to the chest, skin to skin and helping parents maintain a healthy body temperature  **Developer:** Whitelaw and Sleath (1985; based upon the earlier work and recommendation of Peter de Chateau)  **Reference:**  Whitelaw, A., & Sleath, K., (1985). Myth of the Marsupial Mother: Home care of very low birth weight babies in Bogota, Colombia. *Lancet*, *325*(8439), 1206-1208. <https://doi.org/10.1016/S0140-6736(85)92877-6>. | Intervention does not meet inclusion criteria for definition of an attachment-based intervention. This intervention is a developmental neonatal programme for improving preterm infant health development outcomes. |
| **11** | **Incredible Years**  Aims to promote parent emotional and social competence; and to prevent, reduce and treat behavior and emotional problems in children through appropriate boundaries and consequences  **Developer:** Webster-Stratton (circa 1970)  **Reference:** No reference available | Intervention does not meet inclusion criteria for definition of an attachment-based intervention. This intervention focusses on parenting strategies and has a strong social learning theory focus. |
| **12** | **Me, My Baby, Our World**  Aims to provide young parents with a better understanding of their relationship with their baby and the importance of their role in that relationship. It also aims to increase parent’s positive attributions towards their children and increase parental empathy and sensitivity  **Developers:** Stirtzinger et al (2001)  **Reference:**  Stirtzinger, R., McDermid, S., Grusec, J., Bernardini, S., Quinlan, K., & Marshall, M. (2002). Interrupting the inter-generational cycle in high risk pregnancy. *The Journal of Primary Prevention, 23*(1), 7-21. <https://doi.org/10.1023/A:1016535131384>. | This intervention could not be included because there was insufficient information to assess eligibility (author contacted on three occasions). |
| **13** | **Mom2Mom**  Aims to offer support to mothers after the first year of childbirth so that they feel less anxious and depressed and therefore be more sensitive towards their infants needs by providing a stable, secure relationship with a peer  **Developers:** Kaitz et al (2012)  **Reference:** Kaitz, M., Tessler, N., & Chriki, M. (2012). An Attachment-Based Home Visiting Program for Distressed Mothers of Young Infants. *Zero to Three,* 33(2), 43-47. | Intervention does not meet inclusion criteria for definition of an attachment-based intervention. This intervention does not primarily target the caregiver-infant relationship. |
| **14** | **Mothering from the inside out (MIO)**  The programme helps substance using mothers recognise their own emotional stress and the impact this may have on the child. The goals are to improve mother’s emotional regulation and reduce the attachment to substance and enhance wellbeing  **Developers:** Suchman et al (2016)  **Reference:**  Suchman, N.E, Ordway, M.R, de las Heras, L., & McMahon, T.J. (2016). Mothering from the Inside Out: results of a pilot study testing a mentalization-based therapy for mothers enrolled in mental health services. *Attachment & Human Development, 18*(6), 596-617. <https://doi.org/10.1080/14616734.2016.1226371>. | Intervention does not meet inclusion criteria for definition of an attachment-based intervention. This intervention does not primarily target the caregiver-infant relationship. |
| **15** | **New Orleans Intervention Model**  Treatment could include a range of therapeutic interventions, such as: Circle of Security, Parent-Infant Psychotherapy, Attachment and Biobehavioral Catch Up, Parent Child Interaction Therapy, Individual Psychotherapy and Couples Psychotherapy  **Developer:** Minnis (2010; based on the work of Charles Zeanah)  **Reference:**  Minnis, H., Bryce, G., Phin, L., & Wilson, P. (2010). The “Spirit of New Orleans”: Translating a model of intervention with maltreated children and their families for the Glasgow context. *Clinical Child Psychology and Psychiatry*, *15*(4), 497-509. <https://doi.org/10.1177%2F1359104510376124>. | Intervention does not meet inclusion criteria because the developer confirmed that there is no manual available. |
| **16** | **PALME**  Parental training programme for single mothers and their preschool children. The aim is to reduce maternal stressors and improve well-being  **Developer:** Franz et al (2011)  **Reference:**  Franz. M., Weihrauch, L., & Schäfer, R. (2011). PALME: a preventive parental training program for single mothers with preschool aged children. *Journal of Public Health, 19(*4), 305-319. <https://doi.org/10.1007/s10389-011-0396-4>. | Intervention does not meet inclusion criteria due to child target age range is above two years. |
| **17** | **Parent and Child Therapy (PACT)**  Seeks to reframe parent—child relationship distress using narrative techniques and experiential tasks based on the concept of supported looking. Aims to improve parents' reflective capacity and sensitivity, and for the child's sense of security in the primary attachment relationship  **Developers:** Chamber et al (2006)  **Reference:**  Chambers, H., Amos, J., Allison, S., & Roeger, L. (2006). Parent and child therapy: An attachment‐based intervention for children with challenging problems. *Australian and New Zealand Journal of Family Therapy, 27*(2), 68-74. <http://dx.doi.org/10.1002/j.1467-8438.2006.tb00700.x>. | Intervention does not meet inclusion criteria due to child target age range is above two years. |
| **18** | **Parent Child Attachment Play Training**  **(PCAP – Big Toes Little Toes)**  This is a gentle, play-based approach aimed at improving the attachment quality of the parent-child relationship using attachment mechanisms and child-oriented play with parents/carers and their children  **Developer:** Maskell-Graham (2008)  **Reference:** No reference available | Intervention does not meet inclusion criteria due to child target age range is above two years. |
| **19** | **Parent Child Interaction Therapy**  Uses a combination of behavioral therapy, play therapy, and parent training to teach more effective discipline techniques and improve the parent-child relationship where there are problems to communication and poor relations  **Developer:** Eyberg (1988)  **Reference:**  Eyberg, S. (1988). Parent-Child Interaction Therapy. *Child & Family Behavior Therapy*, *10*(1), 33-46. <https://doi.org/10.1300/J019v10n0104>. | Intervention does not meet inclusion criteria due to child target age range is above two years. |
| **20** | **Parent Child Psychotherapy**  Aims to educate the mother about parenting and child development, in order to reduce stress, increase life satisfaction and attachment security  **Developer:** Lieberman (2004)  **Reference:**  Lieberman, A. F. (2004). Child-Parent Psychotherapy: A Relationship-Based Approach to the Treatment of Mental Health Disorders in Infancy and Early Childhood. In A. J. Sameroff, S. C. McDonough, & K. L. Rosenblum (Eds.), Treating parent-infant relationship problems: Strategies for intervention (p. 97–122). Guilford Press. | Intervention does not meet inclusion criteria due to child target age range is above two years. |
| **21** | **Parent Toddler Psychotherapy**  A psychoanalytic intervention targeting mother-infant dyads that may be at risk of an insecure attachment. Aims to prevent or shift an insecure to a secure attachment where mothers are identified as being depressed, anxious, traumatised or at risk of maltreating their child  **Developers:** Cicchetti et al (1999)  **Reference:**  Cicchetti, D., Toth, S. L., & Rogosch, F. A. (1999). The efficacy of toddler-parent psychotherapy to increase attachment security in offspring of depressed mothers. *Attachment and Human* *Development, 1*(1), 34–66. <https://doi.org/10.1080/14616739900134021>. | Intervention does not meet inclusion criteria due to child target age range is above two years. |
| **22** | **Sensory Attachment Intervention**  Uses a child led, constructive play-based approach to support children with a combination of difficulties originating from sensory processing difficulties alongside attachment issues relating to the way that they interact with others, in particular, their primary caregiver  **Developer:** Bhreathnach (2008)  **Reference:** Bhreathnach, É. (2008). *The Scared Gang*. Belfast: Aldertree Press. | This intervention could not be included as there was insufficient information to assess eligibility (author contacted on three occasions). |
| **23** | **Triple P Parenting Programs**  Gives parents simple and practical strategies to help them build strong, healthy relationships, confidently manage their children’s behaviour and prevent problems developing  **Developer:** Sanders (1999)  **Reference:**  Sanders, M.R. (1999). The Triple P-Positive parenting programme: towards an empirically validated multilevel parenting and family support strategy for the prevention of behavior and emotional problems in children. *Clinical Child and Family Psychology Review, 2*(2), 71-90. <https://doi.org/10.1023/a:1021843613840>. | The Triple P interventions do not meet inclusion criteria for definition of an attachment-based intervention. These interventions focus on parenting strategies and have a strong social learning theory focus. |
| ***Interventions without a clear name (listed in alphabetical order of author)*** | | |
|  | **Brief description, author, year and reference** | **Exclusion reason** |
| **1** | Home visiting programme to enhance mother’s capacity to read baby’s signals, respond sensitively and ultimately enhance parent-infant interaction  **Authors:** Ammaniti et al (2006)  **Reference:** Ammaniti, M., Speranza, A. M., Tambelli, R., Muscetta, S., Lucarelli, L., Vismara, L., Odorisio, F. & Cimino, S. (2006). A prevention and promotion intervention program in the field of mother–infant relationship. *Infant Mental Health Journal*, *27*(1), 70-90. <https://doi.org/10.1002/imhj.20081>. | Intervention does not meet inclusion criteria as there is no manual available. |
| **2** | Infant carrying which aims to enhance infant attachment security  **Authors:** Anisfeld et al (1990)  **Reference:** Anisfeld, E., Casper, V., Nozyce, M., & Cunningham, N. (1990). Does infant carrying promote attachment? An experimental study of the effects of increased physical contact on the development of attachment. *Child Development, 61*(5), 1617-1627. <https://doi.org/10.1111/j.1467-8624.1990.tb02888.x>. | Intervention does not meet inclusion criteria as there is no manual available. |
| **3** | Aims to increase reflective functioning and therefore enhance parent-child relationship  **Authors:** Bammens et al (2015)  **Reference:**  Bammens, A. S., Adkins., T & Badger., J. (2015). Psycho-educational intervention increases reflective functioning in foster and adoptive parents. *Adoption and Fostering, 39*(1), 38-50. <https://doi.org/10.1177/0308575914565069>. | Intervention does not meet inclusion criteria as there is no manual available. |
| **4** | Psychotherapeutic programme to improve parental coping and parent-infant attachment through sensitivity training  **Authors:** Brisch et al (2003)  **Reference:**  Brisch, K. H., Bechinger, D., Betzler, S., & Heinemann, H. (2003). Early preventive attachment-oriented psychotherapeutic intervention program with parents of a very low birthweight premature infant: results of attachment and neurological development. *Attachment & Human Development*, *5*(2), 120-135. <https://doi.org/10.1080/1461673031000108504>. | Intervention does not meet inclusion criteria as there is no manual available. |
| **5** | Aim to improve parental mind-mindedness, promote parent-child relationships and reduce child psychopathology  **Authors:** Colonnesi et al (2013)  **Reference:**  Colonnesi, C., Wissink, I.B., Noom, M.J., Asscher, J.J. Hoeve, M., Stams, J.M., Polderman, N., & Kellaert-Knol., M. (2013). Basic Trust: An Attachment-Oriented Intervention Based on Mind-Mindedness in Adoptive Families. *Research on Social Work Practice, 23*(2), 179-188. <https://doi.org/10.1177/1049731512469301>. | Intervention does not meet inclusion criteria as there is no manual available. |
| **6** | Aims to enhance infant attachment through maternal support and infant development information  **Authors:** Jacobson and Frye (1990)  **Reference:** Jacobson, S., & Frye, K. (1990). Effect of maternal social support on attachment: experimental evidence. *Child Development*, 62(3), 572-582. <https://doi.org/10.1111/j.1467-8624.1991.tb01553.x>. | Intervention does not meet inclusion criteria as there is no manual available. |
| **7** | Program for parents with anxiety and/or depression to enhance maternal sensitivity and increasing emotional wellbeing  **Authors:** King et al (2015)  **Reference:**  King, K. L., Priddis, L. E., & Kane, R. T. (2015). Enhancing maternal sensitivity and emotional wellbeing through a preventative parent–child relationship intervention in a community setting. *Journal of Child and Family Studies*, *24*(6), 1582-1592. <http://dx.doi.org/10.1007/s10826-014-9962-z>. | Intervention does not meet inclusion criteria as there is no manual available. |
| **8** | Video-taped and written instruction to enhance maternal responsiveness to the needs of the infant  **Authors:** Lambermon and van Ijzendoorn (1989)  **Reference:**  Lambermon, M.W., & Van Ijzendoorn, M. H. (1989). Influencing mother-infant interaction through videotaped or written instruction: Evaluation of a parent education program. *Early Childhood Research Quarterly*, *4*(4), 449-458. <https://doi.org/10.1016/0885-2006(89)90003-3>. | Intervention does not meet inclusion criteria as there is no manual available. |
| **9** | Combination approach where mother receives psychoeducation, child receives play group therapy and each mother-child dyad receives psychotherapy to enhance attachment security  **Authors:** Nadali et al (2016)  **Reference:**  Nadali, H., Besharat, M.A., Rostami, R., & Bahrami., H.l. (2016). The effectiveness of mother-child attachment-based intervention in enhancing preschoolers' attachment security. *Developmental Psychology: Journal of Iranian Psychologists, 12*(48), 343-354. | Intervention does not meet the inclusion criteria due to child age range is above two years. |
| **10** | Parental sensitivity intervention and confidence/self-efficacy to reduce negative consequences from separation with babies who are born preterm  **Authors:** Phianching et al (2020)  **Reference:**  Phianching, K., Chaimongkol, N., & Pongjaturawit, Y. (2020). Effects of the Parental Sensitivity Intervention Among Mothers and Fathers of Preterm Infants: A Quasi-Experimental Study. *Pacific Rim International Journal of Nursing Research, 24(*2), 246-259. Available from: <https://he02.tci-thaijo.org/index.php/PRIJNR/article/view/195126>. | Intervention does not meet inclusion criteria as there is no manual available. |
| **11** | Psychoanalytic group aiming to address mother’s distress, enhance sensitivity and create containment for infant  **Authors:** Salomonsson et al (2015)  **Reference:**  Salomonsson, M.W., Sorjonen, K., & Salomonsson, B. (2015). A long-term follow-up of a randomized controlled trial of mother-infant psychoanalytic treatment: Outcomes on the children. *Infant Mental Health Journal, 36*(1), 12-29. <http://dx.doi.org/10.1002/imhj.21478>. | Intervention does not meet inclusion criteria as there is no manual available. |
| **12** | Home visiting intervention where there were observations of maternal attachment  **Authors:** Siegel et al (1980)  **Reference:**  Siegel, E., Bauman, K.E., Schaefer, E.S., Saunders, M.M., & Ingram, D.D. (1980). Hospital and home support during infancy: impact on maternal attachment, child abuse and neglect, and health care utilization. *Pediatrics*, 66(2), 183-190. | The article could not be accessed and therefore there was not enough information to assess eligibility. |
| **13** | Mindfulness intervention to address parents attachment style and improve relationships and functioning  **Authors:** Smit et al (2018)  **Reference:**  Smit, S., Martens, C., Ackland, P., & Mikami, A. Y. (2018). Combining attachment and mindfulness to improve family functioning: Pilot of an Attachment-Based Mindfulness program. *Journal of Family Psychotherapy*, *29*(4), 336-358. <https://doi.org/10.1080/08975353.2018.1487247>. | Intervention does not meet inclusion criteria as there is no clear evidence of a manual available. |
| **14** | Aims to address maternal sensitivity, responsiveness and child rearing behaviour to enhance the quality of mother-infant attachment  **Authors:** Torres et al (2011)  **Reference:**  Torres, B., Alonso-Arbiol, I., Cantero, M. J., & Abubakar, A. (2011). Infant-Mother Attachment Can Be Improved through Group Intervention: A Preliminary Evaluation in Spain in a Non-Randomized Controlled Trial. *Spanish Journal of Psychology, 14*(2), 630-638. <https://doi.org/10.5209/rev_SJOP.2011.v14.n2.11>. | Intervention does not meet inclusion criteria as there is no manual available. |
| **15** | Skills based training programme to enhance maternal sensitivity  **Author:** van den Boom (1994)  **Reference:**  van den Boom, D. C. (1994). The influence of temperament and mothering on attachment and exploration: An experimental manipulation of sensitive responsiveness among lower‐class mothers with irritable infants. *Child Development*, *65*(5), 1457-1477. | Intervention does not meet inclusion criteria as there is no manual available. |
| **16** | ‘Babywearing’ (infant carrying) to increase attachment security of infants of adolescent mothers  **Authors:** Williams and Turner (2020)  **Reference:**  Williams, L.R., & Turner, P.R. (2020). Infant carrying as a tool to promote secure attachments in young mothers: Comparing intervention and control infants during the still-face paradigm. *Infant Behavior & Development, 58*. <http://dx.doi.org/10.1016/j.infbeh.2019.101413>. | Intervention does not meet inclusion criteria as there is no manual available. |

**APPENDIX E: Excluded studies (Stage 2), with reasons**

|  | **Author and year** | **Exclusion reason** |
| --- | --- | --- |
| ***Attachment and Biobehavioural Catch Up (ABC)*** | | |
| **1** | Bernard, K., Simons, R., & Dozier, M. (2015). Effects of an attachment‐based intervention on child protective services–referred mothers' event‐related potentials to children's emotions. *Child Development, 86*(6), 1673-1684. <https://doi.org/10.1111/cdev.12418>. | Study does not meet the inclusion criteria because child age range is above two years. |
| **2** | Dozier, M., Peloso, E., Lindhiem, O., Gordon, M. K., Manni, M., Sepulveda, S., Ackerman, J., Bernier, A., & Levine, S. (2006). Developing evidence‐based interventions for foster children: An example of a randomized clinical trial with infants and toddlers. *Journal of Social Issues*, *62*(4), 767-785. <https://doi.org/10.1111/j.1540-4560.2006.00486.x>. | Study does not meet the inclusion criteria because there is no relational measure of the caregiver-infant dyad. |
| **3** | Dozier, M., Lindhiem, O., Lewis, E., Bick, J., Bernard, K., & Peloso, E. (2009). Effects of a foster parent training program on young children’s attachment behaviors: Preliminary evidence from a randomized clinical trial. *Child and Adolescent Social Work Journal*, *26*(4), 321-332. <https://doi.org/10.1007/s10560-009-0165-1>. | Study does not meet the inclusion criteria because the child age range of the sample was not confirmed by the author. |
| **4** | Hepworth, A. D., Berlin, L. J., Martoccio, T. L., Cannon, E. N., Berger, R. H., & Harden, B. J. (2020). Supporting infant emotion regulation through attachment-based intervention: A randomized controlled trial. *Prevention Science*, *21*, 1-12. <https://doi.org/10.1007/s11121-020-01127-1>. | Study does not meet the inclusion criteria because there is no relational measure of the caregiver-infant dyad. |
| **5** | Hepworth, A.D., Berlin, L.J., Salas, K., Pardue-Kim, M., Martoccio, T.L., & Harden, B. (2020). Increasing maternal sensitivity to infant distress through attachment-based intervention: a randomized controlled trial. *Attachment & Human Development*, 1-16. <https://doi.org/10.1080/14616734.2020.1834592>. | Study does not meet the inclusion criteria because the child age range of the sample was not confirmed by the author. |
| **6** | Lind, T., Bernard, K., Ross, E., & Dozier, M. (2014). Intervention effects on negative affect of CPS-referred children: Results of a randomized clinical trial. *Child Abuse & Neglect*, *38*(9), 1459-1467. <https://doi.org/10.1016/j.chiabu.2014.04.004>. | Study does not meet the inclusion criteria because there is no relational measure of the caregiver-infant dyad. |
| **7** | Lind, T., Bernard, K., Yarger, H. A., & Dozier, M. (2020). Promoting compliance in children referred to child protective services: a randomized clinical trial. *Child Development*, *91*(2), 563-576. <https://doi.org/10.1111/cdev.13207>. | Study does not meet the inclusion criteria because the study outcome measures exceed eligible age range. |
| **8** | Zajac, L., Raby, K. L., & Dozier, M. (2020). Sustained effects on attachment security in middle childhood: results from a randomized clinical trial of the Attachment and Biobehavioral Catch‐up (ABC) intervention. *Journal of Child Psychology and Psychiatry*, *61*(4), 417-424. <https://doi.org/10.1111/jcpp.13146>. | Study does not meet the inclusion criteria because the child age range is above two years. |
| **9** | Yarger, H. A., Bernard, K., Caron, E. B., Wallin, A., & Dozier, M. (2020). Enhancing parenting quality for young children adopted internationally: Results of a randomized controlled trial. *Journal of Clinical Child & Adolescent Psychology*, *49*(3), 378-390. <https://doi.org/10.1080/15374416.2018.1547972>. | Study does not meet the inclusion criteria because the study outcome measures exceed eligible age range. |
| **10** | Zimmerman, P.J., Németh, S., & Heinz-Kindler, P. (2021). Promoting Secure Attachment Relationships in Foster Families with the Attachment and Biobehavioral Catch-Up (ABC) Intervention. *Praxis der Kinderpsychologie und Kinderpsychiatrie, 70*(3), 239-254. <http://dx.doi.org/10.13109/prkk.2021.70.3.239>. | Title only. Unable to access. |
| ***Circle of Security (COS)*** | | |
| **1** | Cassidy, J., Brett, B. E., Gross, J. T., Stern, J. A., Martin, D. R., Mohr, J. J., & Woodhouse, S. S. (2017). Circle of Security–Parenting: A randomized controlled trial in Head Start. *Development and Psychopathology*, *29*(2), 651-673. <https://doi.org/10.1017/S0954579417000244>. | Study does not meet the inclusion criteria because the child age range is above two years. |
| **2** | Hoffman, K. T., Marvin, R. S., Cooper, G., & Powell, B. (2006). Changing toddlers' and preschoolers' attachment classifications: The Circle of Security intervention. *Journal of Consulting and Clinical Psychology*, *74*(6), 1017-1026. <https://doi.org/10.1037/0022-006X.74.6.1017>. | Study does not meet the inclusion criteria because the child age range of the sample was not confirmed by the author. |
| **3** | Horton, E., & Murray, C. (2015). A quantitative exploratory evaluation of the circle of security‐parenting program with mothers in residential substance‐abuse treatment. *Infant Mental Health Journal*, *36*(3), 320-336. <https://doi.org/10.1002/imhj.21514>. | Study does not meet the inclusion criteria because the child age range of the sample was not confirmed by the author. |
| **4** | Huber, A., McMahon, C., & Sweller, N. (2015). Improved child behavioural and emotional functioning after Circle of Security 20-week intervention. *Attachment & Human Development*, *17*(6), 547-569. <https://doi.org/10.1080/14616734.2015.1086395>. | Study does not meet the inclusion criteria because the author confirmed that that less than 50% of the child sample was aged 0-2 years. |
| **5** | Huber, A., McMahon, C. A., & Sweller, N. (2015). Efficacy of the 20‐week circle of security intervention: Changes in caregiver reflective functioning, representations, and child attachment in an Australian clinical sample. *Infant Mental Health Journal*, *36*(6), 556-574. <https://doi.org/10.1002/imhj.21540>. | Study does not meet the inclusion criteria because the author confirmed that that less than 50% of the child sample was aged 0-2 years. |
| **6** | Huber, A., McMahon, C., & Sweller, N. (2020). Postintervention and follow-up changes in caregiving behavior and representations after individually or group delivered hybrid Circle of Security-intensive intervention with New Zealand caregiver-child dyads. *Attachment & Human Development*, 1-22. <https://doi.org/10.1080/14616734.2020.1809057>. | Study does not meet the inclusion criteria because the author confirmed that less than 50% of the child sample was aged 0-2 years. |
| **7** | Kitagawa, M., Iwamoto, S., Umemura, T., Kudo, S., Kazui, M., Matsuura, H., Mesman, J. (2021). Attachment-based intervention improves Japanese parent-child relationship quality: A pilot study. *Current Psychology*. 1-11. <https://doi.org/10.1007/s12144-020-01297-9>. | Study does not meet the inclusion criteria because the author confirmed that less than 50% of the child sample was aged 0-2 years. |
| **8** | Maupin, A. N., Samuel, E. E., Nappi, S. M., Heath, J. M., & Smith, M. V. (2017). Disseminating a parenting intervention in the community: Experiences from a multi-site evaluation. *Journal of Child and Family Studies*, *26*(11), 3079-3092. <https://doi.org/10.1007/s10826-017-0804-7>. | Study does not meet the inclusion criteria because the child age range of the sample was not confirmed by the author. |
| **9** | Risholm Mothander, P., Furmark, C., & Neander, K. (2018). Adding “Circle of Security–Parenting” to treatment as usual in three Swedish infant mental health clinics. Effects on parents’ internal representations and quality of parent‐infant interaction. *Scandinavian Journal of Psychology*, *59*(3), 262-272. <https://doi.org/10.1111/sjop.12419>. | Study does not meet the inclusion criteria because the child age range of the sample was not confirmed by the author. |
| **10** | Rose, J., Roman, N., & Mwaba, K. (2018). Circle of Security parenting program efficacy for improving parental self-efficacy in a South African setting: Preliminary evidence. *Journal of Psychology in Africa*, *28*(6), 518-521. <https://doi.org/10.1080/14330237.2018.1523308>. | Study does not meet the inclusion criteria because there is no relational measure of the caregiver-infant dyad included. |
| ***Group Attachment Based Intervention (GABI)*** | | |
| **1** | Murphy, A., Steele, H., Bate, J., Nikitiades, A., Allman, B., Bonuck, K., Meissner, P., & Steele, M. (2015). Group attachment-based intervention. *Family & Community Health*, *38*(3), 268-279. <https://doi.org/10.1097/FCH.0000000000000074>. | Study does not meet the inclusion criteria because there is no relational measure of the caregiver-infant dyad included. |
| ***Mellow Babies*** | | |
| **1** | Puckering, C. (2009). 13 Mellow Babies. *Keeping The Baby in Mind: Infant Mental Health in Practice*, 155. | Does not meet the inclusion criteria because it is not a peer reviewed journal article. |
| ***Mellow Bumps*** | | |
| **1** | White, J., Thompson, L., Puckering, C., Waugh, H., Henderson, M., MacBeth, A., & Wilson, P. (2015). Antenatal parenting support for vulnerable women. *British Journal of Midwifery*, *23*(10), 724-732. <https://doi.org/10.12968/bjom.2015.23.10.724>. | Study does not meet the inclusion criteria because there is no relational measures of the caregiver-infant dyad. |
| ***Mellow Parenting*** | | |
| **1** | Levi, D., Ibrahim, R., Malcolm, R., & MacBeth, A. (2019). Mellow Babies and Mellow Toddlers: Effects on maternal mental health of a group-based parenting intervention for at-risk families with young children. *Journal of Affective Disorders*, *246*(1), 820-827. <https://doi.org/10.1016/j.jad.2018.12.120>. | Study does not meet the inclusion criteria because there is no relational measure of the caregiver-infant dyad included. |
| **2** | Puckering, C., Rogers, J., Mills, M., Cox, A. D., & Raff, M. M. G. (1994). Process and evaluation of a group intervention for mothers with parenting difficulties. *Child Abuse Review*, *3*(4), 299-310. <https://doi.org/10.1002/car.2380030409>. | Study does not meet the inclusion criteria because the author confirmed that it was likely that less than 50% of the child sample was aged 0-2 years. |
| ***Minding the Baby*** | | |
| **1** | Ordway, M. R., Sadler, L. S., Dixon, J., Close, N., Mayes, L., & Slade, A. (2014). Lasting effects of an interdisciplinary home visiting program on child behavior: Preliminary follow-up results of a randomized trial. *Journal of Pediatric Nursing*, *29*(1), 3-13. <https://doi.org/10.1016/j.pedn.2013.04.006>. | Study does not meet the inclusion criteria because the child age range is above two years. |
| ***Mom Power*** | | |
| **1** | Lytle, S., Nagle-Yang, S., Wolfish, E., Gabriel, M., Amin, J., & Huth-Bocks, A. (2018). 5.67 Mom Power©: Promoting Parenting and Maternal Mental Health Among Families With Young Children. *Journal of the American Academy of Child & Adolescent Psychiatry*, *57*(10), S248. | Soes not meet the inclusion criteria because it is a conference abstract. |
| **2** | Muzik, M., Rosenblum, K. L., Alfafara, E. A., Schuster, M. M., Miller, N. M., Waddell, R. M., & Kohler, E. S. (2015). Mom Power: preliminary outcomes of a group intervention to improve mental health and parenting among high-risk mothers. *Archives of Women's Mental Health*, *18*(3), 507-521. <https://doi.org/10.1007/s00737-014-0490-z>. | Study does not meet the inclusion criteria because the child age range of the sample was not confirmed by the author. |
| **3** | Muzik, M., Rosenblum, K., Schuster, M., Kohler, E. S., Alfafara, E., & Miller, N. M. (2016). A mental health and parenting intervention for adolescent and young adult mothers and their infants. *Journal of Depression & Anxiety*, *5*(233), 2167-1044. <https://doi.org/10.4200/2167-1044.1000233>. | Study does not meet the inclusion criteria because there is no relational measure of the caregiver-infant dyad included. |
| **4** | Rosenblum, K. L., Muzik, M., Morelen, D. M., Alfafara, E. A., Miller, N. M., Waddell, R. M., Schuster, M., & Ribaudo, J. (2017). A community-based randomized controlled trial of Mom Power parenting intervention for mothers with interpersonal trauma histories and their young children. *Archives of Women's Mental Health*, *20*(5), 673-686. <https://doi.org/10.1007/s00737-017-0734-9>. | Study does not meet the inclusion criteria because the child age range of the sample was not confirmed by the author. |
| **5** | Rosenblum, K. L., Muzik, M., Morelen, D. M., Alfafara, E. A., Miller, N. M., Waddell, R. M., Schuster, M., & Ribaudo, J. (2017). A community-based randomized controlled trial of Mom Power parenting intervention for mothers with interpersonal trauma histories and their young children. *Archives of Women's Mental Health*, *20*(5), 673-686. DOI: <https://doi.org/10.1007/s00737-017-0734-9>. | Study does not meet the inclusion criteria because there is no relational measure of the caregiver-infant dyad included. |
| **6** | Muzik, M. (2013). Mom power: An attachment-based parenting intervention for trauma-exposed mothers of young children. | Title only. Unable to access – possible conference abstract. |
| **7** | Muzik, M. (2019). Mom Power: A multi-family intervention for mothers with childhood trauma histories and their young children, aimed to support mental health and parenting | Title only. Unable to access – possible conference abstract. |
| ***Mothers and Babies Course (MB)*** | | |
| **1** | Le, H. N., Perry, D. F., & Stuart, E. A. (2011). Randomized controlled trial of a preventive intervention for perinatal depression in high-risk Latinas. Journal of Consulting and Clinical Psychology, 79(2), 135–141. <https://doi.org/10.1037/a0022492>. | Study does not meet the inclusion criteria because there is no relational measure of the caregiver-infant dyad included. |
| **2** | Le, H. N., Perry, D. F., Mendelson, T., Tandon, S. D., & Muñoz, R. F. (2015). Preventing perinatal depression in high risk women: moving the mothers and babies course from clinical trials to community implementation. *Maternal and Child Health Journal*, *19*(10), 2102-2110. <https://doi.org/10.1007/s10995-015-1729-7>. | This study does not meet the inclusion criteria because it is not a peer reviewed journal article. |
| **3** | Le, H. N., Perry, D. F., Villamil Grest, C., Genovez, M., Lieberman, K., Ortiz-Hernandez, S., & Serafini, C. (2021). A mixed methods evaluation of an intervention to prevent perinatal depression among Latina immigrants. *Journal of Reproductive and Infant Psychology*, *39*(4), 382-394. <https://doi.org/10.1080/02646838.2020.1733504>. | Study does not meet the inclusion criteria because there is no relational measure of the caregiver-infant dyad included. |
| **4** | Muñoz, R. F., Le, H. N., Ippen, C. G., Diaz, M. A., Urizar Jr, G. G., Soto, J., & Lieberman, A. F. (2007). Prevention of postpartum depression in low-income women: Development of the Mamás y Bebés/Mothers and Babies Course. *Cognitive and Behavioral Practice, 14*(1), 70-83. <https://doi.org/10.1016/j.cbpra.2006.04.021>. | Study does not meet the inclusion criteria because there is no relational measure of the caregiver-infant dyad included. |
| **5** | Tandon, S. D., Perry, D. F., Mendelson, T., Kemp, K., & Leis, J. A. (2011). Preventing perinatal depression in low-income home visiting clients: A randomized controlled trial. Journal of Consulting and Clinical Psychology, 79(5), 707–712. <https://doi.org/10.1037/a0024895>. | Study does not meet the inclusion criteria because there is no relational measure of the caregiver-infant dyad included. |
| **6** | Tandon, S. D., Leis, J. A., Mendelson, T., Perry, D. F., & Kemp, K. (2014). Six-month outcomes from a randomized controlled trial to prevent perinatal depression in low-income home visiting clients. *Maternal and Child Health Journal*, *18*(4), 873-881. <https://doi.org/10.1007/s10995-013-1313-y>. | Study does not meet the inclusion criteria because there is no relational measure of the caregiver-infant dyad included. |
| **7** | Tandon, S. D., Ward, E. A., Hamil, J. L., Jimenez, C., & Carter, M. (2018). Perinatal depression prevention through home visitation: a cluster randomized trial of mothers and babies 1-on-1. *Journal of Behavioral Medicine*, *41*(5), 641-652. <https://doi.org/10.1007/s10865-018-9934-7>. | Study does not meet the inclusion criteria because there is no relational measure of the caregiver-infant dyad included. |
| ***Mothers and Toddlers Program (MTP)*** | | |
| **1** | Suchman, N., DeCoste, C., Castiglioni, N., Legow, N., & Mayes, L. (2008). The Mothers and Toddlers Program: Preliminary findings from an attachment-based parenting intervention for substance-abusing mothers. *Psychoanalytic Psychology*, *25*(3), 499-517. <https://doi.org/10.1037/0736-9735.25.3.499>. | Study does not meet the inclusion criteria because the child age range of the sample was not confirmed by the author. |
| **2** | Suchman, N. E., DeCoste, C., Castiglioni, N., McMahon, T. J., Rounsaville, B., & Mayes, L. (2010). The Mothers and Toddlers Program, an attachment-based parenting intervention for substance using women: Post-treatment results from a randomized clinical pilot. *Attachment & Human Development*, *12*(5), 483-504. <https://doi.org/10.1080/14616734.2010.501983>. | Study does not meet the inclusion criteria because the child age range of the sample was not confirmed by the author. |
| **3** | Suchman, N. E., Decoste, C., Mcmahon, T. J., Rounsaville, B., & Mayes, L. (2011). The mothers and toddlers program, an attachment‐based parenting intervention for substance‐using women: Results at 6‐week follow‐up in a randomized clinical pilot. *Infant Mental Health Journal*, *32*(4), 427-449. <https://doi.org/10.1002/imhj.20303>. | Study does not meet the inclusion criteria because the child age range of the sample was not confirmed by the author. |
| **4** | Suchman, N. E., Decoste, C., Rosenberger, P., & McMahon, T. J. (2012). Attachment‐based intervention for substance‐using mothers: A preliminary test of the proposed mechanisms of change. *Infant Mental Health Journal*, *33*(4), 360-371. <https://doi.org/10.1002/imhj.21311>. | Study does not meet the inclusion criteria because the child age range of the sample was not confirmed by the author. |
| ***New Beginnings*** | | |
| **1** | Baradon, T., Fonagy, P., Bland, K., Lénárd, K., & Sleed, M. (2008). New Beginnings–an experience-based programme addressing the attachment relationship between mothers and their babies in prisons. *Journal of Child Psychotherapy*, *34*(2), 240-258. <https://doi.org/10.1080/00754170802208065>. | Study does not meet the inclusion criteria because it is a qualitative study. |
| ***Nurturing Attachments*** | | |
| **1** | Gibbons, N., Bacon, A. M., & Lloyd, L. (2019). Is Nurturing Attachments training effective in improving self-efficacy in foster carers and reducing manifestations of Reactive Attachment Disorder in looked after children? *Adoption & Fostering*, *43*(4), 413-428. <https://doi.org/10.1177/0308575919884892>. | Study does not meet the inclusion criteria because the child age range is above two years. |
| **2** | Selwyn, J., Golding, K., Alper, J., Smith, B. G., & Hewitt, O. (2016*). A quantitative and qualitative evaluation of the nurturing attachments group programme*. <https://www.researchgate.net/profile/Julie_Selwyn/publication/309678910_An_evaluation_of_Nurturing_Attachments_-_a_group_programme/links/581ce4c108aeccc08aeca2c6/An-evaluation-of-Nurturing-Attachments-a-group-programme.pdf>. | Study does not meet the inclusion criteria because the child age range is above two years. |
| **3** | Staines, J., Golding, K., & Selwyn, J. (2019). Nurturing attachments parenting program: The relationship between adopters’ parental reflective functioning and perception of their children’s difficulties. *Developmental Child Welfare*, *1*(2), 143-158. <https://doi.org/10.1177/2516103219829861>. | Study does not meet the inclusion criteria because the child age range is above two years. |
| ***Parent Infant Psychotherapy (PIP)*** | | |
| **1** | Cicchetti, D., Rogosch, F. A., & Toth, S. L. (2006). Fostering secure attachment in infants in maltreating families through preventive interventions. *Development and Psychopathology*, *18*(3), 623-649. <https://doi.org/10.10170S0954579406060329>. | Study does not meet the inclusion criteria because the study outcome measures exceed eligible age range. |
| **2** | Dollberg, D., Feldman, R., Tyano, S., & Keren, M. (2013). Maternal representations and mother-infant relational behavior following parent-infant psychotherapy. *Journal of Infant, Child, and Adolescent Psychotherapy*, *12*(3), 190-206. <https://doi.org/10.1080/15289168.2013.821884>. | Study does not meet the inclusion criteria because the child age range of the sample was not confirmed by the author. |
| **3** | Cramer, B., Robert‐Tissot, C., Stern, D. N., Serpa‐Rusconi, S., De Muralt, M., Besson, G., Palacio-Espana, F., Bachmann, J.P., Knauer, D., Berney, C., & D'Arcis, U. (1990). Outcome evaluation in brief mother‐infant psychotherapy: A preliminary report. *Infant Mental Health Journal*, *11*(3), 278-300. [https://doi.org/10.1002/1097-0355(199023)11:3<278::AID-IMHJ2280110309>3.0.CO;2-H](https://doi.org/10.1002/1097-0355(199023)11:3%3c278::AID-IMHJ2280110309%3e3.0.CO;2-H). | Study does not meet the inclusion criteria because the child age range of the sample was not confirmed by the author. It also states a “mother-infant psychotherapy” and the author did not confirm if it is manualised PIP intervention from Stage 1. |
| **4** | Hervé, M. J., Paradis, M., Rattaz, C., Lopez, S., Evrard, V., White-Koning, M., & Maury, M. (2009). Predictors of outcome in infant and toddlers functional or behavioral disorders after a brief parent–infant psychotherapy. *European child & Adolescent Psychiatry*, *18*(12), 737-746. <https://doi.org/10.1007/s00787-009-0032-9>. | Study does not meet the inclusion criteria because there is no relational measure of the caregiver-infant dyad included. |
| **5** | Robert‐Tissot, C., Cramer, B., Stern, D. N., Serpa, S. R., Bachmann, J. P., Palacio‐Espasa, F., Knauer, D., De Mault, M., Berney, C., & Mendiguren, G. (1996). Outcome evaluation in brief mother‐infant psychotherapies: Report on 75 cases. *Infant Mental Health Journal: Official Publication of The World Association for Infant Mental Health*, *17*(2), 97-114. [https://doi.org/10.1002/(SICI)1097-0355(199622)17:2<97::AID-IMHJ1>3.0.CO;2-Y](https://doi.org/10.1002/(SICI)1097-0355(199622)17:2%3c97::AID-IMHJ1%3e3.0.CO;2-Y). | Study does not meet the inclusion criteria because the child age range of the sample was not confirmed by the author. It also states a “mother-infant psychotherapy” and the author did not confirm if it is manualised PIP intervention from Stage 1. |
| **6** | Georg, A. K., Cierpka, M., Schröder-Pfeifer, P., Kress, S., & Taubner, S. (2020). The efficacy of brief Parent−Infant Psychotherapy for treating early regulatory disorders: A randomized-controlled trial. Journal of the American Academy of Child & Adolescent Psychiatry, 60, 723-733. <https://doi.org/10.1016/j.jaac.2020.06.016>. | Study used focused PIP (fPIP) and mentioned a manual. Three citations are given to support fPIP. One was a book wherein the chapter was relevant but there was no direct reference to fPIP and the other two are German publications. |
| **7** | Fonagy, P., Sleed, M., & Baradon, T. (2016). Randomized controlled trial of Parent-Infant Psychotherapy for parents with mental health problems and young infants. Infant Mental Health Journal, 37(2), 97-114. <https://doi.org/10.1002/imhj.21553>. | Study used PIP and mentioned a manual, citing Baradon (2005; author). All authors were affiliated with Anna Freud Centre (specific PIP manual). |
| **8** | Lieberman., A.F., Weston., D.R., & Pawl., J.H. (1991). Preventative intervention and outcome with anxiously attached dyads. Child Development, 62(1), 199-209. | Characterisation of the intervention was not very detailed and there is no mention of a manual. |
| ***The Solihull Approach*** | | |
| **1** | Appleton, R., Douglas, H., & Rheeston, M. (2016). Taking part in ‘Understanding your child’s behaviour’ and positive changes for parents. *Community Practitioner*, *89*(2), 42-8. | Study does not meet the inclusion criteria because there is no relational measure of the caregiver-infant dyad included. |
| **2** | Bateson, K., Delaney, J., & Pybus, R. (2008). Meeting expectations: the pilot evaluation of the Solihull Approach Parenting Group. *Community Practitioner*, *81*(5), 28. | Study does not meet the inclusion criteria because there is no relational measure of the caregiver-infant dyad included. |
| **3** | Brown, S. (2014). Clinical update: a small service evaluation of a Solihull Approach Foster Carer Training Group pilot study. *Practice*, *26*(1), 37-52. <https://doi.org/10.1080/09503153.2013.860094>. | Study does not meet the inclusion criteria because the child age range is above two years. |
| **4** | Douglas, H., & Brennan, A. (2004). Containment, reciprocity and behaviour management: preliminary evaluation of a brief early intervention (the Solihull Approach) for families with infants and young children. *Infant Observation*, *7*(1), 89-107. <https://doi.org/10.1080/13698030408401711>. | Study does not meet the inclusion criteria because there is no relational measure of the caregiver-infant dyad included. |
| **5** | Douglas, H., & Johnson, R. (2019). A randomised controlled trial of the Solihull Approach ten-week group for parents: ‘Understanding your child’s behaviour’(UYCB). *Children*, *5*, 19-2. | Study does not meet the inclusion criteria because the author confirmed that less than 50% of the child sample was aged 0-2 years. |
| **6** | Johnson, R. (2018). Improvements in parenting achieved with innovative online programme: Preliminary evaluation of ‘Understanding Your Child—Online’(UYC-OL): A Solihull Approach course for parents and carers. *Educational and Child Psychology*, *35*(1), 40-50. | Title only. Unable to access. |
| **7** | Madigan, S., Paton, K., & Mackett, N. (2017). The Springfield Project service: evaluation of a Solihull Approach course for foster carers. *Adoption & Fostering*, *41*(3), 254-267. <https://doi.org/10.1177/0308575917719373>. | Study does not meet the inclusion criteria because the child age range is above two years. |
| **8** | Milford, R., Kleve, L., Lea, J., & Greenwood, R. (2006). A pilot evaluation study of the Solihull Approach. *Community Practitioner*, *79*(11), 358. | Study does not meet the inclusion criteria because there is no relational measure of the caregiver-infant dyad included. |
| ***Theraplay*** | | |
| **1** | Eruyar, S., & Vostanis, P. (2020). Feasibility of group theraplay with refugee children in Turkey. *Counselling and Psychotherapeutic Research*, *20*(4), 626– 637. <https://doi.org/10.1002/capr.12354>. | Study does not meet the inclusion criteria because the child age range is above two years. |
| **2** | Siu, A. F. (2009). Theraplay in the Chinese world: An intervention program for Hong Kong children with internalizing problems. *International Journal of Play Therapy*, *18*(1), 1-12. <https://doi.org/10.1037/a0013979>. | Study does not meet the inclusion criteria because the child age range is above two years. |
| **3** | Weir, K. N., Lee, S., Canosa, P., Rodrigues, N., McWilliams, M., & Parker, L. (2013). Whole family theraplay: Integrating family systems theory and theraplay to treat adoptive families. *Adoption Quarterly*, *16*(3-4), 175-200. <https://doi.org/10.1080/10926755.2013.844216>. | Study does not meet the inclusion criteria because the child age range is above two years. |
| **4** | Weir, K. N., Pereyra, S., Crane, J., Greaves, M., Childs, T. S., & Weir, A. B. (2020). The Effectiveness of Theraplay® as a Counseling Practice With Mothers and Their Children in a Substance Abuse Rehabilitation Residential Facility. *The Family Journal*, *29*(1), 115-123. DOI: <https://doi.org/10.1177/1066480720980988>. | Study does not meet the inclusion criteria because the author confirmed that less than 50% of the child sample was aged 0-2 years. |
| **5** | Wettig, H. H. G., Coleman, A. R., & Geider, F. J. (2011). Evaluating the effectiveness of Theraplay in treating shy, socially withdrawn children. International Journal of Play Therapy, 20(1), 26–37. <https://doi.org/10.1037/a0022666>. | Study does not meet the inclusion criteria because the child age range is above two years. |
| ***UCLA Family Development Project*** | | |
| **1** | Heinicke, C. M., Goorsky, M., Levine, M., Ponce, V., Ruth, G., Silverman, M., & Sotelo, C. (2006). Pre‐and postnatal antecedents of a home‐visiting intervention and family developmental outcome. *Infant Mental Health Journal*, *27*(1), 91-119. <https://doi.org/10.1002/imhj.20082>. | Study does not meet the inclusion criteria because it is not a study of an intervention which is included from Stage 1. |

**APPENDIX F: Additional study characteristics of included studies**

|  | **Author(s) and year** | **Random allocation?** | **Random allocation method stated?** | **Power calculation?** | **Reports of blinding** |
| --- | --- | --- | --- | --- | --- |
|  | ***Attachment and Biobehavioral Catch-Up (ABC)*** | | | | |
| **1** | Harden *et al.* (2021) | Yes | No | Not reported | Coders were blind to group allocation |
| **2** | Perrone *et al.* (2020) | Yes | Randomly generated number sequence | Yes | Coders were blind to group allocation |
| **3** | Yarger *et al.* (2019) | Yes | No | Not reported | Coders were blind to intervention status |
| **4** | Berlin *et al.* (2018) | Yes | No | Yes | Coders were blind to group allocation |
| **5** | Caron *et al.* (2016) | N/A | N/A | Not reported | Coders were blind to study hypothesis |
| **6** | Yarger *et al.* (2016) | Yes | No | Not reported | Coders were blind to study condition, intervention session, date of collection and study hypothesis |
| **7** | Berlin *et al.* (2014) | Yes | No | Not reported | Coders were blind to program/control group status |
| **8** | Bick & Dozier (2013) | Yes | No | Not reported | Coders were blind to group assignment of dyads |
| **9** | Bernard *et al.* (2012) | Yes | No | Not reported | Coders were blind to study information |
|  | ***Circle of Security (COS)*** | | | | |
| **1** | Maxwell *et al.* (2020) | No | N/A | Not reported | Not reported |
| **2** | Ramsaeur *et al.* (2019) | Yes | No | Not reported | Maternal sensitivity coders were blind to other information about the mothers from other measures. AAI coders blind to treatment allocation |
| **3** | Cassidy *et al.* (2011) | Yes | No | Not reported | Coders were blind to additional participant information |
| **4** | Cassidy *et al.* (2010) | N/A | N/A | Not reported | Principal coder was blind to the nature of the sample. Videos were intermixed with videos from other high-risk sample groups |
| **5** | Røhder *et al*. (2022) | Yes | Yes | Yes | Randomization was stratified within each municipality. A research assistant generated an allocation list and a person unaffiliated with the project prepared envelopes indicating randomization status from the list. After data were collected at the baseline visit, project personnel opened the envelopes. |
|  | ***Group Attachment-Based Intervention (GABI)*** | | | | |
| **1** | Steele *et al.* (2019) | Yes | Sealed envelope that allocated participants, stratified by age (less than vs greater than 18 months) | Yes | Not reported |
| **2** | Steele *et al.* (2010) | N/A | N/A | Not reported | Coder was blind to maternal AAI status when rating toddler-mother interactions |
| **3** | Myers *et al.* (2022) | N/A | N/A | Not reported | Not reported |
|  | ***Lighthouse MBT Parenting Programme*** | | | | |
| **1** | Byrne *et al.* (2019) | N/A | N/A | Not reported | PDI interviews only were blind coded |
|  | ***Mellow Babies*** | | | | |
| **1** | Puckering *et al.* (2010) | Yes | By the toss of a coin | Not reported | Coders were blind to group status |
| **2** | Raouna et al, (2021) | N/A | N/A | Yes | An independent researcher supervised the  procedure, analysis, and interpretation of the study findings. |
|  | ***Minding the Baby (MTB)*** | | | | |
| **1** | Slade *et al.* (2020) | Yes | Cluster randomisation using the sealed envelope method | Not reported | Coders were blind to group status |
| **2** | Sadler *et al.* (2013) | Yes | Sealed envelope method | Yes | Coders were unaware of group status |
|  | ***Mothers and Babies Course (MB)*** | | | | |
| **1** | Alhusen *et al.* (2020) | Yes | No | Yes (identified as not required due to study design) | Coders were blind to maternal depressive symptoms and conditions assigned |
| **2** | McFarlane *et al.* (2017) | Yes | No | Not reported | Coders were blind to group allocation |
|  | ***New Beginnings*** | | | | |
| **1** | Bain (2014) | Yes | No | Not reported | Coders were unaware of pre or post-intervention status |
| **2** | Sleed *et al.* (2013b) | Yes | Cluster randomisation by an independent statistician | Not reported | Coders were blind to treatment condition and time point |
|  | ***Right from the Start (RFTS)*** | | | | |
| **1** | Niccols (2008) | Yes | No | Not reported | Not reported |
|  | ***Secure Attachment Family Education (SAFE)*** | | | | |
| **1** | Walter *et al.* (2019) | Yes | Block randomisation (block size=10 couples) | Not reported | Coders were unaware of group allocation |
|  | ***Secure Attachment Promotion Program*** | | | | |
| **1** | Santelices *et al.* (2011) | Yes | No | Not reported | Not reported |
|  | ***Strengthening Relationships Towards Secure Attachment*** | | | | |
| **1** | Leigh *et al.* (2013) | N/A | N/A | Not reported | Not reported |
|  | ***The Solihull Approach*** | | | | |
| **1** | Harris-Waller *et al.* (2019) | N/A | N/A | Not reported | Not reported |
| **2** | Douglas & Bateson (2017) | N/A | N/A | Not reported | Not reported |
|  | ***Thula Sana*** | | | | |
| **1** | Valades *et al.* (2021) | Yes | Random number generator used. | Not reported | Coders were blind to group allocation |
| **2** | Cooper *et al.* (2009) | Quasi | Minimisation completed by the trial manager, balancing for depression, whether the pregnancy was planned or where the mother was living | Yes | Coders were unaware of the treatment group |
| **3** | Cooper *et al.* (2002) | N/A | N/A | Not reported | Not reported |
|  | ***UCLA Family Development Project*** | | | | |
| **1** | Heinicke *et al.* (2001) | Yes | No | Not reported | Not reported |
| **2** | Heinicke *et al.* (2000) | N/A | N/A | Not reported | Not reported |
| **3** | Heinicke *et al.* (1999) | Yes | No | Not reported | Not reported |
|  | ***Watch, Wait and Wonder (WWW)*** | | | | |
| **1** | Cohen *et al.* (2002) | Quasi | Two-thirds of the sample were assigned using a table of random numbers, otherwise, the assignment was depended on the caseload and availability of the therapist | Not reported | Group assignment was made by someone who was not involved in the assessment or treatment process |
| **2** | Cohen *et al.* (1999) | Quasi | Two-thirds of the sample were assigned using a table of random numbers, otherwise, the assignment was dependent on the caseload and availability of the therapist | Not reported | Group assignment was made by someone who was not involved in the assessment or treatment process |

**APPENDIX G: Additional measures and findings of included studies**

|  | **Author(s) and year** | **Measures (including timepoint of administration^6^)** | **Main findings (including p-value, if reported)** |
| --- | --- | --- | --- |
| ***Attachment and Biobehavioral Catch Up (ABC)*** | | | |
| **1** | Harden *et al.* (2021) | **CTQ** ^(T1)^  **HITS** ^(T1)^  **CES-D** ^(T1)^  **GAD-7** ^(T1)^  **Observed Toddler Emotional Reactivity**  **BITSEA** ^(T3)^ | ABC group infants showed less behavioural problems when mothers were high-risk compared vs. high-risk control group mothers, *p*=.001* |
| **2** | Perrone *et al.* (2020) | **CESDS**  **ACE-Q** | Post-intervention depression, *p*=.062  Parental ACE moderated intervention mothers depression symptoms and sensitivity |
| **3** | Berlin *et al.* (2018) | **CESDS**^(T1)^ | Depressive symptoms only partially impacted mothers’ parenting style |
| **4** | Caron *et al.* (2016) | Videos of parent-coaches rating the frequency and quality of feedback to parents | Parent-coach fidelity dependent on engagement with supervision and certification |
| **5** | Berlin *et al.* (2014) | **CTQ**^(T1)^  **CESDS**^(T1)^  **GAD-7**^(T1)^ | Prevalence for both groups was high for childhood trauma (95%), depression (67%) and anxiety (33%) |
| ***Circle of Security (COS)*** | | | |
| **1** | Maxwell *et al.* (2020) | **EPDS** | Post-intervention depression symptoms, *p*=<.001*  Change in depression symptoms was dependent on whether parents received the intervention, *p*=.007* |
| **2** | Ramsaeur *et al.* (2019) | **The German version of SCID**^(T1)^  **BDI**^(T1)^  **SCL-90-Revised**^(T1)^ | Mothers in both groups met the diagnostic threshold for diagnosis of depression or comorbid disorder (i.e., anxiety, personality disorder, substance misuse) |
| **3** | Cassidy *et al.* (2011) | **NBAS** | Infants scoring over six were classed as irritable |
| **4** | Cassidy *et al.* (2010) | **SSQ**^(T1)^  **BDI**  **DES**^(T1)^  **RSES**^(T1)^  **TAQ**^(T1)^ | Only depression scores were significantly lower than those taken at enrolment, *p*=.036* |
| **5** | Røhder *et al.* (2022) | **ASQ-SE**  **PRF**  **PSI-SF**  **EPDS**  **SWEMWBS** | Intervention decreased parental stress (*p* = .04)  All other primary and secondary outcome measures were non-significant |
| ***Group Attachment-Based Intervention (GABI)*** | | | |
| **1** | Steele *et al.* (2019) | **ACE-Q**^(T1)^ | Mothers of disorganised attached infants had higher indices of adverse childhood experience than mothers of securely attached infants, *p*=.005* |
| **2** | Steele *et al.* (2010) | **ACE-Q**  **PSI** | There was a high pattern of ACEs within the first 18 years of mother’s lives  Mothers with unresolved attachment reported more parental stress and viewed their infants as more demanding |
| **3** | Myers *et al.* (2022) | **CIB**  **AAI** | Post T2 high spontaneous RF scores were linked to maternal praising, child positive affect and child alertness. Post T3 RF scores were linked to higher maternal elaborating child positive affect. |
| ***Lighthouse MBT Parenting Programme*** | | | |
| **1** | Byrne *et al.* (2019) | **MSES**  **PSI-Short Form**  **PHQ-9**  **GAD-7**  **CORE**  Semi-structured interview on involvement | Post-intervention efficacy*, p*=.018*, parenting stress, *p*=.025*, depression symptoms, *p*=.308, anxiety symptoms, *p*=.222, global distress, *p*=.266  Several parents referred to the intervention as ‘life changing’ and parent feedback reflected significant study findings |
| ***Mellow Babies*** | | | |
| **1** | Puckering *et al.* (2010) | **EPDS** | Post-intervention reported depression symptoms, *p*=.005* |
| **2** | Raouna *et al.* (2021) | **BSI-18**  **KPCS**  **Q-LES-Q-SF**  **ASQ:SE-2**  **Tunnel**  **Feedback** | Program completion associated with significant improvements in anxiety (p=< 0.011, d = 0.27) and overall wellbeing, parenting confidence (p < 0.001, d = 0.46), and perceived closeness of the parent-child relationship. |
| ***Minding the Baby (MTB)*** | | | |
| **1** | Slade *et al.* (2020) | **CESDS**^(T1)^  **MPTSD-Civilian Form**^(T1)^ | Post-intervention depression symptoms, *p*=.07*; positive associations across groups between baseline depressive symptoms and 24 months depressive symptoms  Positive associations found across both groups for baseline depressive symptoms and 24 months PTSD and baseline PTSD and 24 months PTSD |
| **2** | Sadler *et al.* (2013) | **CESDS**^(T1, T3, T4)^  **BSI-Short Form**^(T1, T3, T4)^  Clinical interview and health record review^(T3, T4)^ | No significant group difference in psychopathology symptoms  Intervention group was more likely than the control group to be up to date with immunisations at 12 months, but both groups were up to date by 24 months, *p*=.019* |
| ***Mothers and Babies Course (MB)*** | | | |
| **1** | Alhusen *et al.* (2020) | **EPDS** ^(T1, T2, T3)^ | Higher average reduction in depressive symptoms scores for MB mothers vs. control group: 5.77 vs. 2.50 |
| **2** | McFarlane *et al.* (2017) | **BDI-II**  **WOC Questionnaire**  **LES**  **PSS** | Post-intervention reduction in depression, *p*=.05*, *d*=.38; and stress, *p*=.06*, *d*=.35  Post-intervention increased coping, *p* <.01*, *d* =.55  There were no significant differences on the LES |
| ***New Beginnings*** | | | |
| **1** | Bain (2014) | **Griffiths Scales (Luiz *et al.*, 2006)**  **Kessler-10 (Kessler *et al.*, 2002)** | Post-intervention effect of group attendance on infant’s speech development, *p*=.025*; mean speech delay decreasing overtime for intervention mothers  Post-intervention levels of depression and anxiety: 72% reported higher symptom levels |
| **2** | Sleed *et al.* (2013b) | **CESDS** | No significant group differences between depression overtime or between depression levels |
| ***Right from the Start (RFTS)*** | | | |
| **1** | Niccols (2008) | Five-point scale of cooperation, involvement and likeability | Participants highly valued the intervention and many reported being more confident with caring for their baby (86-93%)  RFTS cost significantly less than the treatment as usual control group, *p*=.001* |
| ***Secure Attachment Family Education (SAFE)*** | | | |
| **1** | Walter *et al.* (2019) | **TAQ**^(T1)^  **PSDS**^(T1)^  **BDI**^(T1)^  **Zweierbeziehungsbogen Questionnaire (Cierpka & Frevert, 1994)^(^**^T1)^  Open-ended questions categorised with motivation to participate^(T2)^ | More fathers in the control group reported past abuse, *p*=.005*  High rate of participation from fathers (79.7%) in the SAFE intervention. Mothers and fathers were rated as participating equally in both groups |
| ***The Solihull Approach*** | | | |
| **1** | Harris-Waller *et al.* (2019) | **SDQ**  **Carer Questionnaire (developed by Kim Golding, n.d.)**  **PSI- Short Form** | Post-intervention hyperactivity and inattention, *p*=.02*; understanding their infant’s difficulties, *p*=.007*; understanding their infant’s behaviour, *p*=.03*; skills to manage their infant’s difficulties, *p*=.004*  Overall decrease in parental stress, but did not reach significance |
| **2** | Douglas & Bateson (2017) | **PHQ-4**  **PRAQ**  **Intention to breastfeed rated with five-point multiple questions**  **Intention to stop smoking with five-point multiple questions** | No significant differences for anxiety, *p*=.667; depression, *p*=.071; intention to stop smoking, *p*=.109  Post-intervention intention to breastfeed, *p*=.005*  Pregnancy-related anxiety was lower for mothers, *p*=.001* but not fathers, *p*=.135 |
| ***Thula Sana*** | | | |
| **1** | Cooper *et al.* (2009) | **SCID**^(T1, T2)^ | Six months depression symptoms*, p*=.36  12 months depression symptoms, *p*=.82 |
| **2** | Cooper *et al.* (2002) | **Anthropometric measures of the infant**  **SCID**  **Questionnaire using four-point multiple choice** | Post-intervention infant weight, *p*=.01*; length, *p*=.02*  Post-intervention depression vs. control: 19% vs. 28%  Overall ratings were positive approximately 90% rated intervention highly |
| ***UCLA Family Development Project*** | | | |
| **1** | Heinicke *et al.* (2001) | **BSID^(^**^T5^**^)^:** development and performance subscales  **BDI**^(T1-T5)^  **CSS**^(T1)^  **SAS**^(T1)^  **MAT**^(T1)^  **Maternal support interview**^(T1-T5)^**:** perceived quality of partner support and perceived quality of family support | Post-intervention infant mental development, *p*=.830; performance development, *p*=.334  Post-intervention depression and anxiety, *p*=.787  Six months post-intervention partner and family support, *p*=.05*  12 months post-intervention partner and family support, *p*=.006*  24 months post-intervention partner and family support, *p*=.001* |
| **2** | Heinicke *et al.* (2000) | **PDE**  **Maternal support interview:** perceived quality of partner support and perceived quality of family support  **62 items rated at each home visit on positive connection, trust and work with intervenor** | A lower number of visits accurately reflected a lower level of maternal involvement.  Correlates with a higher number of visits and positive connection, *p*=.0003*, trust, *p*=.0001*; work, *p*=.0003* |
| **3** | Heinicke *et al.* (1999) | **BSID**^(^**^T5^**^)^**:** performance and development subscales  **BDI**^(^**^T1-T5^**^)^  **CSS**^(^**^T1^**^)^  **SAS**^(^**^T1^**^)^  **MAT**^(^**^T1^**^)^  **Maternal support interview**^(^**^T1-T5)^:** perceived quality of partner support and perceived quality of family support  **WAIS**^(^**^T1^**^)^ | Post-intervention improvements in infant mental development and performance development, caregiver depression and anxiety but group differences not significant  Post-intervention partner and family support, *p*=.16* |
| ***Watch, Wait and Wonder (WWW)*** | | | |
| **1** | Cohen *et al.* (2002) | 100-point rating scale of problem behaviours  **BSID:** mental subscales  **PSCS**  **PSI**  **BDI** | (Problem behaviours reported in 1999 study)  Follow-up comfort dealing with infant behaviour, *p*=.01*; parenting stress, *p*=.05*; infant emotion regulation and development were maintained  Control group decreased in emotion regulation, *p*=.05* and development, *p*=.05*  Follow-up depression symptoms reduced in both groups |
| **2** | Cohen *et al.* (1999) | 100-point rating scale of problem behaviours  **BSID:** mental subscales  **PSCS**  **PSI**  **BDI**  **The Working Alliance Inventory (Hovarth & Greenberg, 1986)** | The most common presenting problems were sleeping/feeding (63.6%), behavioural regulation (36.4%), attachment (42.4%), parenting (24.2%) and development (15.2%)  Post-intervention development, *p*=.05*; emotion regulation, *p*=.05*; caregiver competence, *p*=.01*; depression symptoms, *p*=.01*  Mothers in both groups reported less stress associated with parenting and their infant, *p*=.001*  No significant group difference in working alliance with the therapist |

^6^Time point identified if difference to that of design; *statistically significant finding; ACE-Q= Adverse Childhood Experiences Questionnaire (Felitti *et al.*, 1998); ASQ= Ages and Stages Questionnaire (Squires *et al.*, 2002); BDI= Beck Depression Inventory (Beck, 1978); BITSEA= Brief Infant Toddler Social Emotional Assessment (Briggs-Gowan *et al.*, 2004); BSI= Brief Symptom Inventory (Derogatis, 1993); BSID= Bayley Scales of Infant Development (Bayley, 1969); CESDS= Center for Epidemiological Studies Depression Scale (Radloff, 1977); CORE= Clinical Outcome in Routine Evaluation Scale (Evans *et al.*, 2006); CSS= Cutrona Support Scale (Cutrona, 1984); CTQ= Childhood Trauma Questionnaire (Bernstein & Frink, 1998); DES= Dissociative Experiences Scale (Carlson & Putnam, 1993); EPDS= Edinburgh Postnatal Depression Scale (Cox & Holden, 1994); GAD= Generalized Anxiety Disorder 7-Item Scale (Spitzer *et al.*, 2006); HITS= Hurts, Insults, Threatens and Screams (Sherin *et al.*, 1998); LEI= Life Event Inventory (Egeland *et al.*, 1979); LES= Life Experience Survey (Sarson *et al.*, 1978); MAT= Locke-Wallace Marital Adjustment Test (Locke & Wallace, 1959); MPTSD= Mississippi Scale for Assessment of PTSD (Keane *et al.*, 1988); MSES= Maternal Self-Efficacy Scale (Teti & Gelfand, 1991); NBAS= Neonatal Behavioural Assessment Scale (Brazelton & Nugent, 1995); PBI= Parental Bonding Instrument (Parker *et al.*, 1979); PDE= Personality Disorder Evaluation (Loranger, 1988); PHQ= Personal Health Questionnaire (Kroenke *et al.*, 2009); PRAQ= Pregnancy Related Anxiety Questionnaire (Rini *et al.*, 1991); PSCS= Parenting Sense Competence Scale (Johnston & Marsh, 1991); PSDS= Posttraumatic Stress Diagnostic Scale (Foa *et al.*, 1997); PSI= Parenting Stress Index (Abdin, 1995; Troster, 2011); PSS= Perceived Stress Scale (Cohen *et al.*, 1983); RSES= Rosenberg Self-Esteem Scale (Rosenberg, 1979); SAS= Speilberger Anxiety Scale (Speilberger, 1977); SCID= Structured Clinical Interview for DSM-IV Diagnosis (First *et al.*, 1996; Fydrich *et al.*, 1997); SCL= Symptom Checklist (Schmidt *et al.*, 1989; Franke, 2002); SDQ= Strength and Difficulties Questionnaire (Goodman, 1997); SMS= Self-Mastery Scale (Pearlin & Schooler, 1978); SSQ= Social Support Questionnaire (Sarason *et al.*, 1983); TAQ= Traumatic Antecedents Questionnaire (van der Kolk, 1997); WAIS= Weschler Adult Intelligence Scale (Weschler, 1981); WOC= Ways of Coping Questionnaire (Edwards & O’Neill, 1998).

**References (Appendix E)**

Abidin, R. R. (1995). *Parenting stress index: Professional manual* (3rd ed.). Psychological Assessment Resources.

Bayley, N. (1969). *Manual for the Bayley scales of infant development*. Psychological Corporation.

Beck, A.T. (1978). *Beck depression inventory*. Centre for Cognitive Therapy.

Bernstein, D.P., & Fink, L., (1998). *CTQ childhood trauma questionnaire: A retrospective self-report manual*. NCS Pearson.

Brazelton, T. B., & Nugent, J. K. (1995). *The neonatal behavioral assessment scale.* McKeith Publishers.

Briggs-Gowan, M. J., Carter, A. S., Irwin, J. R., Wachtel, K., & Cicchetti, D. V. (2004). The brief infant-toddler social and emotional assessment: Screening for social-emotional problemsand delays in competence. *Journal of Pediatric Psychology, 29*(2), 143–155. <https://doi.org/10.1093/jpepsy/jsh017>.

Carlson, E.B., & Putnam, F.W. (1993). An update on the dissociative experiences scale. *Dissociation: Progress in the Dissociative Disorders, 6*(1), 16–27.

Cierpka, M., & Frevert, G. (1994). Die familienbögen: Ein inventar zur einschätzung von familienfunktionen. Hogrefe, Verlag f. Psychologie.

Cohen, S., Kamarck, T., & Mermelstein, R. (1983). A global measure of perceived stress. *Journal of* *Health and Social Behavior, 24*(4), 385–396. <https://doi.org/10.2307/2136404>.

Cox, J. & Holden, J. (1994). *Perinatal psychiatry: Uses and abuses of the Edinburgh postnatal depression scale.* Gaskell Publishers.

Cutrona, C.E. (1984). Social support and stress in the transition to parenthood. *Journal of Abnormal* *Psychology, 93*(4), 378–390. <https://doi.org/10.1037/0021-843X.93.4.378>.

Derogatis, L.R. (1993). *The brief symptom inventory*. National Computer Systems.

Edwards, J. R., & O'Neill, R. M. (1998). The construct validity of scores on the Ways of Coping Questionnaire: Confirmatory analysis of alternative factor structures. *Educational and Psychological Measurement*, *58*(6), 955-983. <https://doi.org/10.1177%2F0013164498058006007>.

Egeland, B., Deinard, A., Brunnquell, D., Phipps-Yonas, S., & Crichton, L. (1979). *A prospective study of the antecedents of child abuse* [final report. Grant No. 90-C-424]. University of Minnesota

Evans, C., Mellor-Clark, J., Margison, F., Barkham, M., Audin, K., Connell, J., & McGrath, G. (2000). CORE: Clinical outcomes in routine evaluation. *Journal of Mental Health*, *9*(3), 247–255. <https://doi.org/10.1080/jmh.9.3.247.255>.Felitti, V. J., Anda, R. F., Nordenberg, D., Williamson, D. F., Spitz, A. M., Edwards, V., & Marks, J. S. (1998). Relationship of childhood abuse and household dysfunction to many of the leading causes of death in adults: The adverse childhood experiences (ACE) study. *American Journal of Preventive Medicine, 14*(4), 245–257. <https://doi.org/10.1016/S0749-3797(98)00017-8>.

First, M. B., Spitzer, R. L., Gibbon, M., & Williams, J. B. W. (1996). *Structured clinical interview for DSM-IV axis I disorders, clinician version (SCID-CV)*. American Psychiatric Press.

Foa, E. B., Cashman, L., Jaycox, L., & Perry, K. (1997). The validation of a self-report measure of posttraumatic stress disorder: The posttraumatic diagnostic scale. *Psychological Assessment, 9*(4), 445–451. <https://doi.org/10.1037/1040-3590.9.4.445>.

Franke, G. H. (2002). SCL-90-R. Symptom checkliste von L. R. Derogatis. Deutsche Version. Zweite, vollständig überarbeitete und neu normierte Auflage. Beltz Test Gmbh.

Fydrich, T., Renneberg, B., Schmitz, B., & Wittchen, H. U. (1997). SKID-P. Strukturiertes klinisches interview für DSM-IV achse II, Persönlichkeitsstörungen. Hogrefe.

Greenspan, S. I., & Wieder, S. (1984). Dimensions and levels of the therapeutic process. *Psychotherapy: Theory, Research, Practice, Training, 21*(1), 5-23. <https://doi.org/10.1037/h0087529>.

Groß, S., Reck, C., Thiel-Bonney, C., & Cierpka, M. (2013). Empirische grundlagen des fragebogens zum schreien, feuttern und schlafen (SFS). *Praxis der Kinderpsychologie und Kinderpsychiatrie, 62*(5), 327-347. <https://doi.org/10.13109/prkk.2013.62.5.327>.

Goodman, R. (1997). The strengths and difficulties questionnaire: A research note. J*ournal of Child Psychology and Psychiatry, 38*(5), 581–586. <https://doi.org/10.1111/j.1469-7610.1997.tb01545.x>.

Horvath, A.O., & Greenberg, L.S. (1986). The development of the working alliance inventory. In L.S. Greenberg & W.M. Pinsof (Eds.), *The psychotherapeutic process: A research handbook* (pp. 529–556). Guilford Press.

Johnston, C., & Mash, E.J. (1991). A measure of parenting satisfaction and efficacy. *Journal of Clinical Child Psychology, 18*(2), 167–175. <https://doi.org/10.1207/s15374424jccp1802_8>.

Keane, T., Caddell, J., & Taylor, K. L. (1988). Mississippi scale for combat-related PTSD: Three studies in reliability and validity. *Journal of Consulting and Clinical Psychology, 56*(1), 85–90. <https://doi.org/10.1037/0022-006X.56.1.85>.

Kessler, R.C., Andrews, G., Colpe, L.J., Hiripi, E., Mroczek, D.K., Norman, S.L., Walters, E.E., & Zaslavsky., A.M. (2002). Short screening scales to monitor population prevalences and trends in non-specific psychological distress. *Psychological Medicine, 32*(6), 959–956. [https://doi.org/10.1017}S0033291702006074](https://doi.org/10.1017%7dS0033291702006074).

Kroenke, K., Spitzer Robert, L., & Williams Janet, B. W. (2001). The PHQ-9. *Journal of General Internal Medicine*, *16*(9), 606–613. <https://doi.org/10.1046/j.1525-1497.2001.016009606.x>.

Locke, H., & Wallace, K. (1959). Short marital adjustment and prediction tests: Their reliability and validity. *Marriage and Family Living, 21*(3), 251–255. <https://doi.org/10.2307/348022>.

Loranger, H.W. (1988). *Personality disorder examination (PDE) manual*. DV Communications.

Luiz,D., Faragher, B., Barnard, A., Knoesen, N., Kotras, N., Burns, L.E., & Challis, D. (2006). *Griffiths mental developmental scales-extended revised: 2 to 8 years analysis manual*. Hogrefe.

Papoušek, M., Rothenburg, S., Cierpka, M., & Hofacker, N. V. (2006). Regulationsstörungen der frühen kindheit. CD-basierte fortbildung, Stiftung Kindergesundheit, München.

Parker, G., Tupling, H., & Brown, L.P. (1979). A parental bonding instrument*. British Journal of Medical Psychology, 52*(1), 1–10. <https://doi.org/10.1111/j.2044-8341.1979.tb02487.x>.

Pearlin, L. I., & Schooler, C. (1978). The structure of coping. *Journal of Health and Social Behavior, 19*(1), 2-21. <https://doi.org/10.2307/2136319>.

Sherin, K., Sinacore, J., Li, X., Zitter, R., & Shakil, A. (1998). HITS: A short domestic violence screening tool for use in a family practice setting. *Family Medicine, 30*, 508–512. <https://www.researchgate.net/profile/Kevin-Sherin/publication/13616105_HITS_A_short_domestic_violence_screening_tool_for_use_in_a_family_practice_setting/links/02e7e538a4f3a20d28000000/HITS-A-short-domestic-violence-screening-tool-for-use-in-a-family-practice-setting.pdf>.

Radloff, L. S. (1977). The CES-D scale. *Applied Psychological Measurement, 1*(3)*,* 385–401. <http://dx.doi.org/10.1177/014662167700100306>.

Rini, C. K., Dunkel-Schetter, C., Wadhwa, P. D., & Sandman, C. A. (1999). Psychological adaptation and birth outcomes: The role of personal resources, stress, and sociocultural context in pregnancy. *Health Psychology*, *18*(4), 333-345. <https://doi.org/10.1037/0278-6133.18.4.333>.

Rosenberg, M. (1979). *Conceiving the self.* Basic Books.

Sarason, I., Johnson, J., & Siegel, J. (1978). Assessing the impact of life changes: Development of the life experiences survey. *Journal of Consulting and Clinical Psychology, 46*(5), 932–946. <https://psycnet.apa.org/doi/10.1037/0022-006X.46.5.932>.

Sarason, B.R., Shearin, E.N., Pierce, G.R., & Sarason, I.G. (1987). Interrelations of social support measure: Theoretical and practical implications. *Journal of Personality and Social Psychology, 52*(4), 813–832. <https://doi.org/10.1037/0022-3514.52.4.813>.

Schmidt, J., Lamprecht, F., & Wittmann, W. W. (1989). Satisfaction with inpatient management. Development of a questionnaire and initial validity studies. *Psychotherapie, Psychosomatik, Medizinische Psychologie*, *39*(7), 248-255.

Spitzer. R.L., Kroenke, K., Williams, J.B.W., & Lowe, B. (2006). A brief measure for assessing generalized anxiety disorder. *Archives of Internal Medicine, 166*(10), 1092–1097. <https://doi.org/doi:10/1001/archinte166.10.1092>.

Spielberger, C.D. (1977). *Self-evaluation questionnaire*. Consulting Psychologists Press.

Squires, J., Bricker, D., Heo, K., & Twombly, E. (2002). *Ages & stages questionnaires: Social-emotional. A parent-completed, child-monitoring system for social-emotional behaviors*. Brookes Publishing.

Teti, D. M., & Gelfand, D. M. (1991). Behavioral competence among mothers of infants in the first year: The mediational role of maternal self-efficacy. *Child Development*, *62*(5), 918–929. <https://doi.org/10.1111/j.1467-8624.1991.tb01580.x>.

Troster, H. (2011). *Eltern-belastungs-inventar: EBI; Deutsche version des parenting stress index* *(PSI) von RR Abidin*. Gottingen, Hogrefe.

van der Kolk, B. (1997). *Trauma assessment package: Traumatic antecedents questionnaire (TAQ)*. Trauma Center.

Wechsler, D. (1981). *Wechsler adult intelligence scale-revised*. Psychological Corp.
